# Mobilise-D insights to estimate real-world walking speed in multiple conditions with a wearable device

**DOI:** 10.1038/s41598-024-51766-5

**Published:** 2024-01-19

**Authors:** Cameron Kirk, Arne Küderle, M. Encarna Micó-Amigo, Tecla Bonci, Anisoara Paraschiv-Ionescu, Martin Ullrich, Abolfazl Soltani, Eran Gazit, Francesca Salis, Lisa Alcock, Kamiar Aminian, Clemens Becker, Stefano Bertuletti, Philip Brown, Ellen Buckley, Alma Cantu, Anne-Elie Carsin, Marco Caruso, Brian Caulfield, Andrea Cereatti, Lorenzo Chiari, Ilaria D’Ascanio, Judith Garcia-Aymerich, Clint Hansen, Jeffrey M. Hausdorff, Hugo Hiden, Emily Hume, Alison Keogh, Felix Kluge, Sarah Koch, Walter Maetzler, Dimitrios Megaritis, Arne Mueller, Martijn Niessen, Luca Palmerini, Lars Schwickert, Kirsty Scott, Basil Sharrack, Henrik Sillén, David Singleton, Beatrix Vereijken, Ioannis Vogiatzis, Alison J. Yarnall, Lynn Rochester, Claudia Mazzà, Bjoern M. Eskofier, Silvia Del Din, Mobilise-D consortium, Francesca Bottin, Lorenzo Chiari, Cristina Curreli, Ilaria D’Ascanio, Giorgio Davico, Roberta De Michele, Giuliano Galimberti, Luca Palmerini, Saverio Ranciati, Luca Reggi, Marco Viceconti, Lucia D’Apote, Jules Desmond, Megan Doyle, Mary Elliot-Davey, Gilles Gnacadja, Anja Kassner, Beat Knusel, Monika Pocrzepa, Nicolas Pourbaix, Hoi-Shen Radcliffe, Lening Shen, Jennifer Simon, Jesper Havsol, Diana Jarretta, Magnus Jornten-karlsson, Pierre Mugnier, Solange Corriol Rohou, Gabriela Saraiva, Henrik Sillén, Michael Boettger, Igor Knezevic, Frank Kramer, Paolo Piraino, Hubert Trübel, Hajar Ahachad, Hubert Blain, Sylvie Broussous, Francois Canovas, Florent Cerret, Louis Dagneaux, Valerie Driss, Florence Galtier, Charlote Kaan, Stephanie Miot, Eva Murauer, Anne-Sophie Vérissimo, Daniela Berg, Kirsten Emmert, Clint Hansen, Hanna Hildesheim, Jennifer Kudelka, Walter Maetzler, Corina Maetzler, Christian Schlenstedt, Valdo Arnera, Karin Beckstrom, Patrick Folaron, Antonia Gizdic, Fay Horak, Skender Imeri, Stefanie Krieger, Narcis Nica, Natalia Pletneva, Stephen Raymond, Donna Reed, Ara Sekaram, Kristen Sowalsky, Kamiar Aminian, Anisoara Ionescu, Abolfazl Soltani, Bjoern Eskofier, Felix Kluge, Arne Küderle, Martin Ullrich, Victoria Alcaraz Serrano, Magda Bosch de Basea, Joren Buekers, Gabriela Cardenas, Anne-Elie Carsin, Ines Cobo, Anna Delgado Llobet, Laura Delgado Ortiz, Mariona Font Garcia, Judith Garcia Aymerich, Elena Gimeno-Santos, Alicia Jose, Sarah Koch, Ashar Ahmad, Marcel Froehlich, Gilyana Borlikova, Marie-Sidonie Edieux, Ronan Fox, Bill Holt, Kellee Howard, Sean Kelly, Sheila Kelly, Ruth Lalor, Alexandre Malouvier, Kusuma Manavalli Ramanna, Marie Mc Carthy, Gerard Quinn, Isaac Rodriguez Chavez, Peter Schueler, Michal Skackov, Barbara Skerrit, Sara Buttery, Nicholas Hopkinson, Alexis Perkins, Keir Philip, Mike Polkey, Parris Williams, Michael Jackson, David Wenn, Sofie Breuls, Heleen Demeyer, Nitesh Ghosh, Pieter Ginis, Lies Glorie, Valerie Haerens, Lova Hulst, Femke Hulzinga, Wim Janssenns, Alice Nieuwboer, Thierry Troosters, Tim Vanhoutte, Myriam Witvrouw, Marieke Wuyts, Luca Cornelisse, Jordi Evers, Siete Frouws, Neall Mouthaan, Martijn Niessen, Laura Siepman, Aida Aydemir, Yann Hyvert, Martin Aursand Berge, Mara Diaconu, Monika Engdal, Karoline Blix Grønvik, Jorunn Helbostad, Lars Gunnar Johnsen, Anna Marcuzzi, Ingalill Midtsand, Mari Odden, Ingvild Saltvedt, Erika Skaslien, Kristin Taraldsen, Beatrix Vereijken, Ola Bunte, Wim Dartee, Gul Erdemli, Olivier Grenet, Tilo Hache, Sam Hariry, Sabina Hernandez Penna, Felix Kluge, Jacek Lukawy, Suzanne Maahs, Ram Miller, Arne Mueller, Jens Praestgaard, Ronenn Roubenoff, Sandra Schluechter, Leen van Steenbergen, Xuemei Cai, Charmaine Demanuele, Charmaine Demanuele, Mariana Gameiro, Di Junrui, Isik Karahanoglu, Joe Mather, Dimitrios Psaltos, Emma Stokes, Anil Tarachandani, Hao Zhang, Anne-Marie Kirsten, Kirsten Paash, Martina Russ, Henrik Watz, Ines Zimmermann, Clemens Becker, Niki Brenner, Christoph Endress, Martha Gierka, Clarissa Huber, Simon Jaeger, Carl-Philipp Jansen, Bernd Kinner, Jochen Klenk, Elena Litz, Elena Litz, Stefanie Mikolaizak, Kilian Rapp, Matthias Schwab, Lars Schwickert, Erkin Uysal, Martin Wohlrab, Vanessa Zoller, Nadir Ammour, Stephanie Bascle, Fabrice Bonche, Manon Cariou, Matthieu Jouannin, Mike Chambers, Antonella Ciucchiuini, Ariel Dowling, Emilio Merlo-Pich, Max Tolkoff, Lucy Fry, Mark Gordon, Pippa Loupe, Michal Melamed, Michael Reich, Sara Shnider, Marina Brozgol, David Buzaglo, Pablo Cornejo Thumm, Eran Gazit, Nir Giladi, Jeff Hausdorff, Talia Herman, Inbar Hillel, Anat Mirelman, Ayala Saban, Shahar Yehezkyahu, Nikolaos Chynkiamis, Stefano Bertuletti, Marco Caruso, AndreaCereatti, Andrea Manca, Francesca Salis, Valeria Bonanno, Giampaolo Brichetto, Gloria Dalla Costa, Comi Giancarlo, Letizia Leocani, Allia Mahajneh, Matteo Martinis, Mariaemma Rodegher, Andrea Tacchino, Mauro Zaffaroni, Mauro Zaffaroni, Gilbert Buesching, Anja Frei, Katharina Hackl, Melanie Keller, Marion Maggi-Beba, Ashley Polhemus, Milo Puhan, Thomas Riegler, Thomas Sigrist, Sabine Spielmanns, Marc Spielmanns, Valerie Zumbrunnen, Stafanie Dettmer, Heiko Gassner, Teresa Greinwalder, Konstantin Huhn, Jelena Jukic, Jochen Klucken, Franz Marxreiter, Florian Nickel, Martin Regensburger, Veit Rothhammer, Sarah Seifferth, Sabine Stallforth, Tanja Stirnweiß, Andrea Weitzenfelder, Juergen Winkler, Antonio Bevilaqua, Brian Caulfield, Cathy Goulding, Georgiana Ifrim, Tahar Kechadi, Alison Keogh, Brian Mac Namee, Milu Philip, David Singleton, Lisa Alcock, Graham Armitage, Jaume Bacardit, Harry Bailey, Phil Brown, Alma Cantu, Laura Cordova-Rivera, Silvia Del Din, Brook Galna, Ann Gibson, Ashley Hart, Hugo Hiden, Chloe Hinchliffe, Sara Johansson Fernstad, Cameron Kirk, Ellen Lirani-Silva, Encarna Micó Amigo, Isabel Neatrour, Emma Packer, Annette Pantall, Jian Qing Shi, Lynn Rochester, Emily Hume, Dimitrios Megaritis, Ioannis Vogiatzis, Sarah Birchall, Tecla Bonci, Gavin Brittain, Ellen Buckley, Fabio Ciravegna, Sooji Han, Liam Haslam, Neil Ireson, Azza Ishmail, Mahjabin Islam, Vita Lanfranchi, Michael Long, Claudia Mazzà, Jessica McNeil, Shagun Misraq, Sarah Moll, Ahmed Mubarak-Mohamed, Siva Nair, David Paling, Shivani Patel, Dibya Pattanaik, Daisy Priest, Alex Radford, Kirsty Scott, Basil Sharrack, Lubos Vaci, Linda Van Gelder

**Affiliations:** 1 Translational and Clinical Research Institute, Faculty of Medical Sciences, Newcastle University, The Catalyst 3 Science Square, Room 3.27, Newcastle Upon Tyne, NE4 5TG UK; 2 Machine Learning and Data Analytics Lab, Department Artificial Intelligence in Biomedical Engineering, Friedrich-Alexander-Universität Erlangen-Nürnberg, Erlangen, Germany; 3 Department of Mechanical Engineering and Insigneo Institute for in Silico Medicine, The University of Sheffield, Sheffield, UK; 4 Laboratory of Movement Analysis and Measurement, Ecole Polytechnique Federale de Lausanne, Lausanne, Switzerland; 5 Center for the Study of Movement, Cognition and Mobility, Neurological Institute, Tel Aviv Sourasky Medical Center, Tel Aviv, Israel; 6 Department of Biomedical Sciences, University of Sassari, Sassari, Italy; 7 National Institute for Health and Care Research (NIHR) Newcastle Biomedical Research Centre (BRC), Newcastle University and the Newcastle Upon Tyne Hospitals NHS Foundation Trust, Newcastle Upon Tyne, UK; 8 Robert Bosch Gesellschaft für Medizinische Forschung, Stuttgart, Germany; 9 Department of Electronics and Telecommunications, Politecnico di Torino, Turin, Italy; 10 The Newcastle Upon Tyne Hospitals NHS Foundation Trust, Newcastle Upon Tyne, UK; 11 School of Computing, Newcastle University, Newcastle Upon Tyne, UK; 12 Barcelona Institute for Global Health (ISGlobal), Barcelona, Spain; 13 Universitat Pompeu Fabra, Barcelona, Catalonia Spain; 14 CIBER Epidemiología y Salud Pública (CIBERESP), Madrid, Spain; 15 Insight Centre for Data Analytics, University College Dublin, Dublin, Ireland; 16 School of Public Health, Physiotherapy and Sports Science, University College Dublin, Dublin, Ireland; 17 Department of Electrical, Electronic and Information Engineering «Guglielmo Marconi», University of Bologna, Bologna, Italy; 18 Health Sciences and Technologies—Interdepartmental Center for Industrial Research (CIRI-SDV), University of Bologna, Bologna, Italy; 19 Department of Neurology, University Medical Center Schleswig-Holstein Campus Kiel, Kiel, Germany; 20 Department of Physical Therapy, Sagol School of Neuroscience, Sackler Faculty of Medicine, Tel Aviv University, Tel Aviv, Israel; 21 Rush Alzheimer’s Disease Center and Department of Orthopaedic Surgery, Rush University Medical Center, Chicago, IL USA; 22 Department of Sport, Exercise and Rehabilitation, Northumbria University Newcastle, Newcastle Upon Tyne, UK; 23 Novartis Institutes of Biomedical Research, Novartis Pharma AG, Basel, Switzerland; 24 McRoberts BV, The Hague, The Netherlands; 25 Department of Neuroscience and Sheffield NIHR Translational Neuroscience BRC, Sheffield Teaching Hospitals NHS Foundation Trust, Sheffield, UK; 26 Digital Health R&D, AstraZeneca, Sweden; 27 Department of Neuromedicine and Movement Science, Norwegian University of Science and Technology, Trondheim, Norway; 28 Alma Mater Studiorum - Università di Bologna, Bologna, Italy; 29 Amgen, Thousand Oaks, CA USA; 30 AstraZeneca AB, Gothenburg, Sweden; 31 Bayer Aktiengesellschaft, Leverkusen, Germany; 32 Centre Hospitalier Universitaire de Montpellier, Montpellier, France; 33 Christian-Albrechts-Universität, Kiel, Germany; 34 Clario, Philadelphia, USA; 35 Ecole Polytechnique Federale de Lausanne, Lausanne, Switzerland; 36 Friedrich-Alexander-Universitaet Erlangen-Nuernberg, Erlangen, Germany; 37 Fundacion Privada Instituto de Salud Global, Barcelona, Spain; 38 Gruenenthal GMBH, Aachen, Germany; 39 ICON Clinical Research Limited, Dublin, Ireland; 40 Imperial College of Science Technology and Medicine, London, UK; 41 Ixscient Ltd, London, UK; 42 Katholieke Universiteit Leuven, Leuven, Belgium; 43 McRoberts B.V., The Hague, The Netherlands; 44 Merck KGaA, Darmstadt, Germany; 45 Norges Teknisk-Naturvitenskapelige Universitet, Trondheim, Norway; 46 Novartis Pharma AG, Basel, Switzerland; 47 Pfizer Limited, Tadworth, UK; 48 Pneumologisches Forschungsinstitut an der LungenClinic Grosshansdorf GmbH, Großhansdorf, Germany; 49 Robert Bosch Gesellschaft Fur Medizinische Forschung MBH, Stuttgart, Germany; 50 Sanofi Aventis Recherche et Developpement, Chilly-Mazarin, France; 51 Takeda, Tokyo, Japan; 52 Teva Pharmaceutical Industries Ltd, Tel Aviv-Yafo, Israel; 53 The Foundation for Medical Research Infrastructural Development and Health Services, Tel Aviv-Yafo, Israel; 54 Thorax Foundation, Athens, Greece; 55 Università Degli Studi di Sassari, Sassari, Italy; 56 Università Vita-Salute San Raffaele, Milan, Italy; 57 Universitat Zurich, Zurich, Switzerland; 58 Universitatsklinikum Erlangen, Erlangen, Germany; 59 University College Dublin, Dublin, Ireland; 60 University of Newcastle, Newcastle Upon Tyne, UK; 61 University of Northumbria, Newcastle Upon Tyne, UK; 62 University of Sheffield, Sheffield, UK

**Keywords:** Biomarkers, Biomedical engineering, Outcomes research, Medical research, Translational research

## Abstract

This study aimed to validate a wearable device’s walking speed estimation pipeline, considering complexity, speed, and walking bout duration. The goal was to provide recommendations on the use of wearable devices for real-world mobility analysis. Participants with Parkinson’s Disease, Multiple Sclerosis, Proximal Femoral Fracture, Chronic Obstructive Pulmonary Disease, Congestive Heart Failure, and healthy older adults (n = 97) were monitored in the laboratory and the real-world (2.5 h), using a lower back wearable device. Two walking speed estimation pipelines were validated across 4408/1298 (2.5 h/laboratory) detected walking bouts, compared to 4620/1365 bouts detected by a multi-sensor reference system. In the laboratory, the mean absolute error (MAE) and mean relative error (MRE) for walking speed estimation ranged from 0.06 to 0.12 m/s and − 2.1 to 14.4%, with ICCs (Intraclass correlation coefficients) between good (0.79) and excellent (0.91). Real-world MAE ranged from 0.09 to 0.13, MARE from 1.3 to 22.7%, with ICCs indicating moderate (0.57) to good (0.88) agreement. Lower errors were observed for cohorts without major gait impairments, less complex tasks, and longer walking bouts. The analytical pipelines demonstrated moderate to good accuracy in estimating walking speed. Accuracy depended on confounding factors, emphasizing the need for robust technical validation before clinical application.

*Trial registration*: ISRCTN – 12246987.

## Introduction

Mobility has been defined as the ability to move freely and easily, representing an essential component of health and quality of life, being key to physical, mental, and social well-being^1^. Sudden loss in mobility has been associated with morbidity, falls, dementia, cognitive decline, hospitalizations, mortality and symptoms of chronic disorders^2–6^. Mobility loss translates to an inability to perform activities of daily living, which has also been defined as mobility disability^1,7^. According to the World Health Organization (WHO), it is estimated that 1 billion people currently live with mobility disability due to impairments in their respiratory, cardiovascular, musculoskeletal or neurological systems^8^. This number is expected to rise further due to increased longevity of the worldwide population and prolonged survival in people with chronic diseases leading to motor detriment. This will entail large public health implications and increase an already growing social and economic burden upon healthcare systems.

Efforts to mitigate the loss of mobility disability are of increasing priority and have been the focus of several recent clinical intervention trials^9–12^. Existing mobility endpoints are based upon patient self-reporting and one-off assessments of physical function, such as the timed up and go test or a 6 minutes walk test. While such an assessment provides useful insights on an individual’s mobility capacity (how much they can do), it lacks ecological validity as it does not reflect an individual’s mobility performance (how much they actually do) during daily life^1,7,13,14^. This incomplete assessment of mobility limits therapeutic development and clinical management^14^. Therefore, valid and easy-to-use methods to accurately and reliably assess mobility performance would provide insight into how mobility disability manifests in the real-world^7^.

Digital health technology, such as wearable devices, offer an objective, low-cost and ecologically valid approach of continuously monitoring real-world mobility performance through characterisation of Digital Mobility Outcomes (DMOs)^7^. A single wearable device can be worn unobtrusively and comfortably on the lower back, attached to a belt or affixed to the skin^15,16^. Walking speed remains the most widely explored DMO^17^, where reduced walking speed has been associated with ageing, mortality, neurological and cardiovascular conditions, cognitive decline, and risk of falling^2,5,18–21^. Furthermore, walking speed represents a composite measure of walking ability, as it is estimated from the combination of other spatial and temporal DMOs, specifically stepping cadence and stride length. As such, walking speed represents a global measure of mobility that can be interpreted and is meaningful to patients and clinicians alike^17^.

A lack of robust technical validation of real-world walking speed measurements has prevented the adoption of walking speed derived from wearable devices as a clinical endpoint for interventional trials^7,22,23^. Technical validation requires the comparison of DMOs quantified from a wearable device with DMOs quantified by an established reference system, whilst accounting for and acknowledging a wide range of contextual factors^24^. The majority of algorithms to estimate walking speed have typically been validated based upon healthy adults assessed in simple laboratory tasks in standardized and supervised settings that do not represent the more challenging and variable nature of real-world environments^24^. Furthermore, studies often validate DMO algorithms in isolation, without considering the complexity of a comprehensive multi-stage pipeline needed to estimate walking speed^25–28^. This first requires the identification of walking activity, followed by the quantification of DMOs (e.g., steps, cadence, stride length, walking speed). Interactions between all algorithmic steps in the pipeline will influence final outputs, and errors will accumulate along the pipeline. A robust validation of walking speed therefore requires analysis of the estimate of walking speed at a walking bout (WB) level from the implementation of the full pipeline.

The aim of this study was to provide a comprehensive validation of walking speed estimated from a single inertial measurement unit based wearable device against a multi-sensor reference system integrating pressure insoles (INDIP)^29–31^, to enable a robust validation: (i) in both laboratory and real-world settings, (ii) across different clinical cohorts with a range of mobility disabilities, (iii) across gait tasks of varying complexity and (iv) accounting for confounding factors (WB duration and walking speed). Subsequently, we provide recommendations for the suitability of a wearable device paired with the proposed analytical pipeline as a measure of real-world mobility and suggest a framework for future studies aiming to validate DMOs for real-world monitoring.

## Results

Clinical and demographic characteristics of the participants are presented by cohort in Table 1 (Mean ranges: Age 47–79 years, Height 166–176 cm, mass 70.6–83.6 kg). Participants were recruited (n = 108) from the following cohorts: congestive heart failure (CHF), chronic obstructive pulmonary disease (COPD), healthy adult (HA), multiple sclerosis (MS), Parkinson’s disease (PD) and proximal femoral fracture (PFF). Eleven participants (CHF: 4, MS: 3, PD: 1, PFF: 3) were excluded from the laboratory recordings and 26 participants (CHF: 3, HA: 3, MS: 7, PD: 5, PFF: 8) from the real-world recordings due to technical difficulties with either the reference system or the wearable device.

**Table 1:** Demographic and clinical characteristics of the participants included in the real-world analysis.

Characteristic	HA (n = 17)	CHF (n = 9)	COPD (n = 17)	MS (n = 13)	PD (n = 15)	PFF (n = 11)
Age (years)	72.35 ± 6.00	68.00 ± 12.80	69.35 ± 9.10	47.23 ± 11.09	69.20 ± 7.48	79.70 ± 6.86
Height (cm)	167.00 ± 10.91	176.33 ± 8.75	168.97 ± 6.61	166.31 ± 9.11	172.73 ± 7.96	170.23 ± 9.07
Weight (kg)	74.36 ± 12.53	83.61 ± 19.56	73.71 ± 14.22	80.09 ± 22.11	79.13 ± 16.27	70.59 ± 16.86
Walking aid users (% [n])	5% [1]	22% [2]	5% [1]	30% [4]	26% [4]	45% [5]
MoCa [0–30]	28.18 ± 1.38	26.56 ± 3.21	24.65 ± 3.39	26.23 ± 3.49	23.93 ± 4.45	25.09 ± 4.46
Hoehn & Yahr stage (n)					H&Y I: 3 H&Y II: 7 H&Y III: 5	
MDS-UPDRS III [0–132]					30.67 ± 13.33	
EDSS [0–10]				3.85 ± 1.72		
SPPB [0–12]						7.73 ± 3.10
CAT Score [0–40]			19.65 ± 8.95			
FEV1/FVC			0.54 ± 0.12			
6MWT distance (m)		348.81 ± 164.34	357.65 ± 88.52			
KCCQ-12 Score [0–100]		82.39 ± 18.51				

Values are presented as mean ± standard deviation.

*CAT* chronic obstructive pulmonary disease (COPD) assessment test, *EDSS* expanded disability status scale, *FEV1* forced expiratory volume in 1 second, *KCCQ-12* Kansas City cardiomyopathy questionnaire-12, *MDS-UPDRS III* Movement disorder society unified Parkinson’s disease rating scale part III, *MoCA* montreal cognitive assessment, *SPPB* Short physical performance battery, *6MWT* 6 minute walking test, *HA* healthy adults, *PD* Parkinson’s disease, *MS* multiple sclerosis, *COPD* chronic obstructive pulmonary disease, *CHF* congestive heart failure, *PFF* proximal femoral fracture.

**Figure 1:**
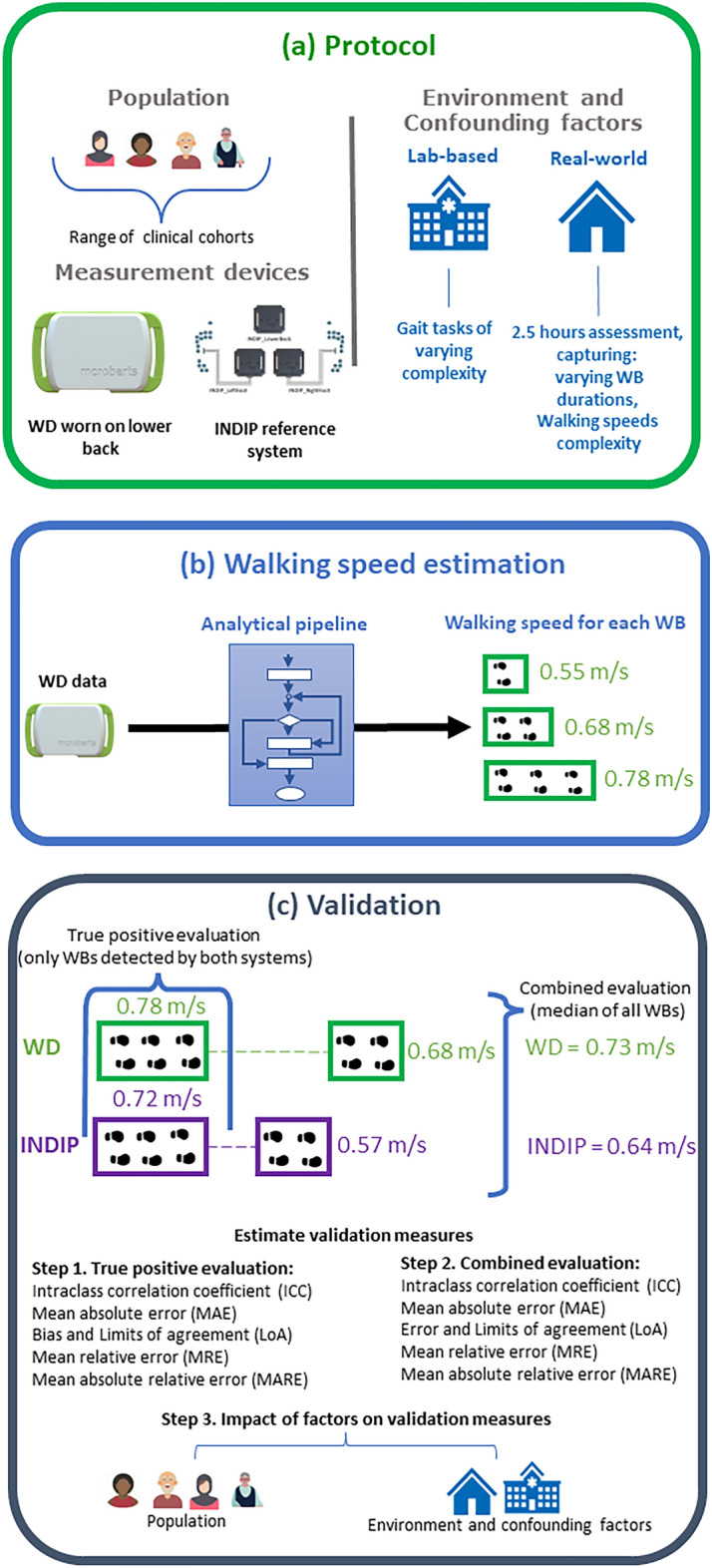
Overview of (**a**) the TVS protocol, (**b**) the analytical pipeline applied to estimate walking speed from the wearable device data (WD), (**c**) the approach to validating walking speed estimated from the analytical pipeline.

An overview of the method of validating walking speed can be viewed in Fig. 1.

### True Positive Evaluation

**Figure 2:**
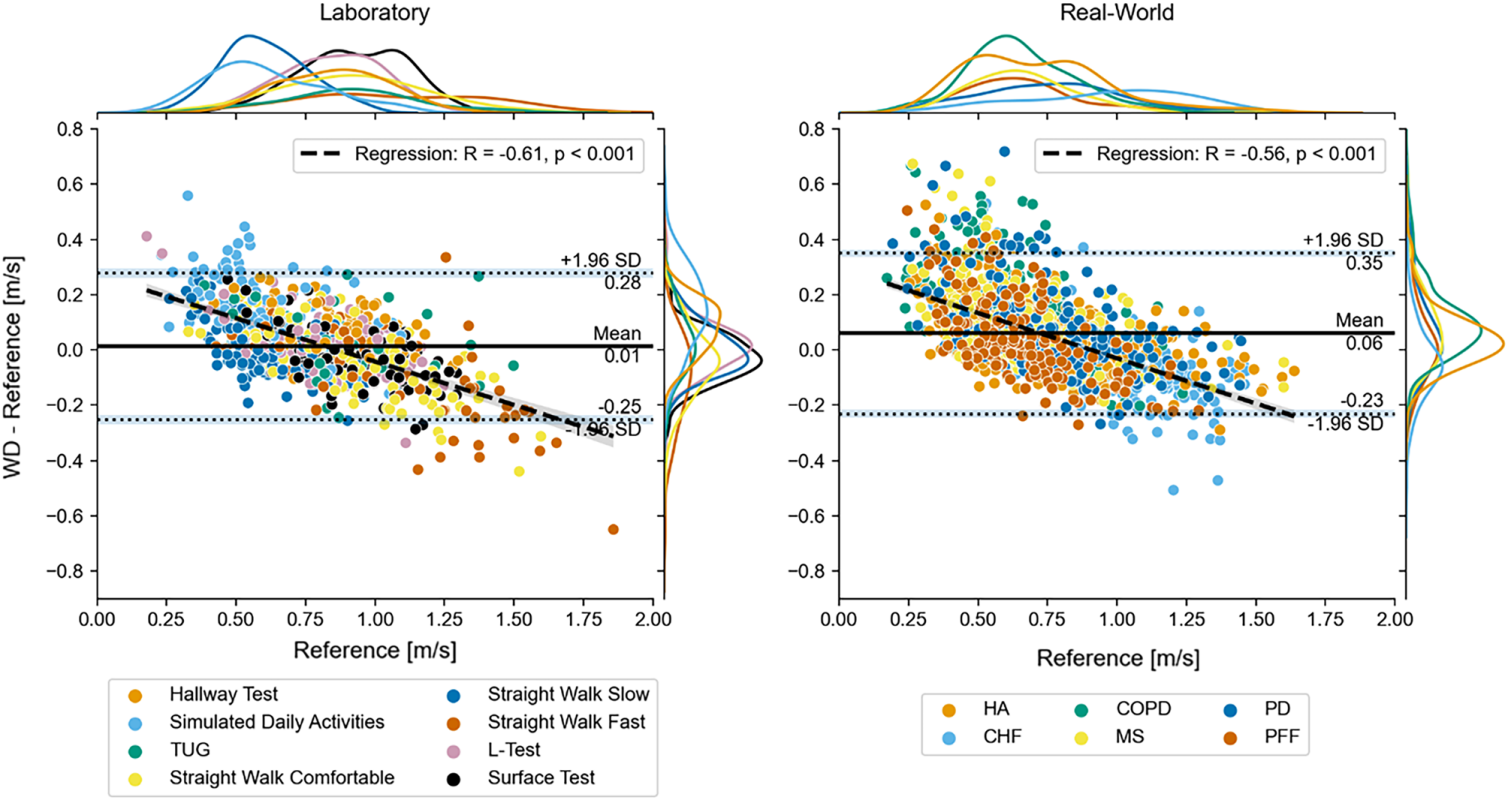
Residual plots of walking speed for all true-positive WBs recorded in the laboratory (left) and during the real-world recording (right). The margin plots represent the overall speed and error distributions. The margin plots are further grouped by the performed tests for the laboratory and by the cohort for the real-world recordings. The light blue bars around the Limits of Agreement (LOA) (dashed horizontal lines) represent their bootstrapped confidence intervals. The dashed black line represents the result of a linear regression on all datapoints. The grey area around the regression line represents the bootstrapped 95% confidence intervals.

In the laboratory assessments a total of 1365 WBs were detected by the reference system and 1298 WBs by the wearable device. To be able to compare DMOs on a WB level, the analysis included WBs that were concurrently detected by both systems (true positive analysis). All WBs with a time-overlap of more than 80% of their duration were considered true positive, resulting in 692 WBs that were considered for analysis and considered a TP (see Methods and Supplementary Figs. 1 and 2 for more details). Based on these true positive WBs, we observed a mean error of 0.01 m/s (MRE = 5.9%), and MAE of 0.10 m/s (MARE = 14.96%) across all cohorts (Fig. 2, left. Table 2). We found that walking speed was estimated with good reliability (ICC = 0.84) by the wearable device, with a slight overestimation compared to the INDIP reference system*.*

**Table 2:** characterization of relative and absolute errors, Intraclass correlation coefficient (ICC), Limits of agreement (LoA), for walking speed estimated from the true-positive walking bouts (WBs) from all Laboratory tasks combined and the real-world assessment.

Cohort	n-WBs (#)	Reference (m/s)	Wearable device (m/s)	Error with LOA (m/s)	Rel. error with LOA (%)	Abs error (m/s)	Rel. Abs. error (%)	ICC
Laboratory								
HA	103	0.86 [0.54, 1.15]	0.86 [0.54, 1.17]	− 0.00 [− 0.21, 0.20]	0.55 [− 28.19, 29.28]	0.08 [0.01, 0.21]	10.34 [0.68, 29.22]	0.86 [0.80, 0.90]
CHF	60	0.94 [0.45, 1.52]	0.88 [0.56, 1.29]	− 0.06 [− 0.37, 0.26]	− 2.06 [− 40.71, 36.60]	0.12 [0.01, 0.30]	13.86 [1.72, 37.90]	0.82 [0.72, 0.89]
COPD	106	0.89 [0.59, 1.13]	0.90 [0.58, 1.14]	0.01 [− 0.15, 0.17]	2.27 [− 20.62, 25.16]	0.06 [0.01, 0.16]	7.82 [0.75, 19.35]	0.91 [0.87, 0.94]
MS	169	0.83 [0.45, 1.27]	0.85 [0.53, 1.18]	0.02 [− 0.26, 0.30]	6.58 [− 38.09, 51.25]	0.11 [0.01, 0.28]	15.87 [1.20, 48.71]	0.81 [0.75, 0.86]
PD	133	0.80 [0.47, 1.20]	0.82 [0.54, 1.15]	0.02 [− 0.26, 0.31]	7.99 [− 45.21, 61.19]	0.11 [0.01, 0.33]	17.01 [1.28, 45.27]	0.79 [0.72, 0.85]
PFF	121	0.70 [0.35, 1.34]	0.74 [0.44, 1.12]	0.04 [− 0.26, 0.33]	14.43 [− 50.98, 79.83]	0.12 [0.01, 0.32]	22.19 [1.19, 66.49]	0.82 [0.75, 0.87]
All	692	0.83 [0.43, 1.28]	0.84 [0.52, 1.18]	0.01 [− 0.25, 0.28]	5.92 [− 40.94, 52.77]	0.10 [0.01, 0.28]	14.96 [0.93, 46.15]	0.84 [0.81, 0.86]
Real-world								
HA	364	0.72 [0.38, 1.22]	0.76 [0.47, 1.23]	0.04 [− 0.18, 0.27]	10.53 [− 35.92, 56.98]	0.09 [0.01, 0.26]	15.45 [0.83, 56.00]	0.88 [0.85, 0.90]
CHF	172	0.95 [0.46, 1.38]	0.92 [0.56, 1.33]	− 0.04 [− 0.35, 0.27]	1.29 [− 48.12, 50.70]	0.12 [0.01, 0.32]	15.48 [1.24, 53.42]	0.82 [0.76, 0.86]
COPD	328	0.65 [0.36, 1.01]	0.76 [0.51, 1.06]	0.11 [− 0.16, 0.38]	22.71 [− 43.84, 89.26]	0.13 [0.01, 0.40]	24.88 [1.05, 91.11]	0.57 [0.49, 0.64]
MS	196	0.67 [0.40, 1.02]	0.76 [0.53, 1.06]	0.09 [− 0.20, 0.39]	19.56 [− 48.42, 87.53]	0.13 [0.01, 0.37]	23.53 [1.70, 80.64]	0.63 [0.54, 0.71]
PD	192	0.76 [0.37, 1.19]	0.84 [0.52, 1.20]	0.08 [− 0.24, 0.40]	17.79 [− 52.94, 88.53]	0.13 [0.01, 0.38]	22.99 [0.64, 94.50]	0.72 [0.64, 0.78]
PFF	162	0.66 [0.38, 1.04]	0.69 [0.45, 0.97]	0.04 [− 0.25, 0.32]	10.63 [− 50.73, 71.98]	0.11 [0.01, 0.33]	20.02 [1.24, 73.39]	0.66 [0.56, 0.74]
All	1414	0.72 [0.38, 1.21]	0.78 [0.49, 1.18]	0.06 [− 0.23, 0.35]	14.48 [− 47.17, 76.13]	0.11 [0.01, 0.36]	20.31 [0.95, 76.20]	0.77 [0.75, 0.79]

Values are either provided as mean and [5%, 95%] quantile or as mean and limit of agreement, if indicated by LoA.

In the 2.5-h real-world assessment, the reference system detected 4409 WBs, while the wearable device identified 4620 WBs. The average sensitivity and specificity for WB detection compared to the reference system were 0.65 and 0.99, respectively (Table 3). Across all detected WBs, 1414 (30% of all WBs) were identified as true positive WBs (i.e., more than 80% overlap with a reference WB). Based on these WBs, we observed a mean error of 0.06 m/s (MRE = 14.48%) and a MAE of 0.11 m/s (MARE = 20.31%) across all cohorts (Fig. 2, right, Table 2). As observed in the laboratory data, results showed a good reliability (ICC = 0.77) (Table 4), with an overestimation of walking speed by the wearable device (0.01 m/s).

**Table 3:** The performance of the WB detection calculated by comparing, sample by sample, the detected walking bout regions by the single wearable device with the detected walking bout regions by the reference system in the real-world recordings.

Cohort	Accuracy	Sensitivity	Specificity	Positive Predictive Value
HA	0.93 [0.87, 0.98]	0.72 [0.57, 0.91]	0.99 [0.98, 1.00]	0.97 [0.92, 0.99]
CHF	0.89 [0.74, 0.97]	0.57 [0.49, 0.68]	0.99 [0.97, 1.00]	0.97 [0.92, 1.00]
COPD	0.94 [0.89, 0.98]	0.67 [0.55, 0.82]	0.98 [0.96, 1.00]	0.89 [0.71, 0.99]
MS	0.95 [0.88, 0.99]	0.67 [0.46, 0.89]	0.99 [0.97, 1.00]	0.90 [0.72, 0.99]
PD	0.92 [0.72, 0.99]	0.60 [0.33, 0.82]	0.99 [0.93, 1.00]	0.93 [0.72, 1.00]
PFF	0.92 [0.85, 0.99]	0.61 [0.47, 0.83]	0.99 [0.98, 1.00]	0.96 [0.88, 1.00]
All	0.93 [0.83, 0.99]	0.65 [0.45, 0.87]	0.99 [0.97, 1.00]	0.93 [0.79, 1.00]

Performance values are first calculated per participant and then aggregated per cohort, over all participants. Results are provided as mean and confidence intervals.

**Table 4:** Walking speed ranges and error analysis for the results combined per Laboratory test (laboratory) or over the entire real-world recording.

Cohort	n-DPs (#)	Reference (m/s)	Wearable device (m/s)	Error with LoA (m/s)	Rel. error with LoA (%)	Abs. error (m/s)	Rel. Abs. Error (%)	ICC
Laboratory								
HA	136	0.90 [0.56, 1.32]	0.85 [0.57, 1.15]	− 0.05 [− 0.34, 0.23]	− 3.65 [− 34.63, 27.33]	0.12 [0.01, 0.30]	12.72 [0.86, 32.94]	0.74 [0.66, 0.81]
CHF	58	0.92 [0.45, 1.52]	0.87 [0.52, 1.24]	− 0.05 [− 0.40, 0.30]	− 0.54 [− 41.45, 40.38]	0.13 [0.01, 0.38]	14.42 [0.90, 39.66]	0.80 [0.68, 0.87]
COPD	109	0.88 [0.47, 1.23]	0.87 [0.55, 1.13]	− 0.01 [− 0.34, 0.31]	1.51 [− 32.13, 35.16]	0.10 [0.01, 0.27]	11.65 [0.60, 35.76]	0.72 [0.61, 0.80]
MS	129	0.81 [0.43, 1.29]	0.84 [0.52, 1.23]	0.03 [− 0.26, 0.33]	9.05 [− 38.46, 56.55]	0.11 [0.01, 0.28]	16.92 [0.74, 60.84]	0.81 [0.74, 0.86]
PD	132	0.79 [0.42, 1.27]	0.82 [0.53, 1.20]	0.03 [− 0.25, 0.32]	9.10 [− 38.67, 56.86]	0.11 [0.01, 0.31]	17.25 [1.35, 52.02]	0.82 [0.76, 0.87]
PFF	119	0.65 [0.23, 1.24]	0.72 [0.43, 1.04]	0.07 [− 0.24, 0.37]	26.05 [− 60.69, 112.78]	0.14 [0.01, 0.32]	32.57 [1.84, 124.21]	0.80 [0.73, 0.86]
All	683	0.81 [0.40, 1.31]	0.82 [0.51, 1.18]	0.01 [− 0.31, 0.32]	7.47 [− 47.71, 62.66]	0.12 [0.01, 0.30]	17.82 [0.75, 59.08]	0.80 [0.77, 0.83]
Real-world								
HA	17	0.56 [0.44, 0.76]	0.61 [0.51, 0.71]	0.05 [− 0.13, 0.24]	11.89 [− 21.95, 45.74]	0.09 [0.03, 0.16]	17.25 [3.67, 32.29]	0.41 [− 0.06, 0.73]
CHF	9	0.66 [0.42, 0.84]	0.82 [0.66, 1.04]	0.17 [− 0.02, 0.35]	29.62 [− 12.61, 71.86]	0.17 [0.02, 0.26]	29.80 [2.04, 61.60]	0.45 [− 0.21, 0.84]
COPD	17	0.59 [0.47, 0.72]	0.66 [0.52, 0.78]	0.07 [− 0.07, 0.21]	12.67 [− 14.18, 39.52]	0.08 [0.01, 0.17]	14.12 [1.98, 32.21]	0.43 [− 0.04, 0.75]
MS	13	0.57 [0.40, 0.74]	0.71 [0.57, 0.83]	0.13 [− 0.15, 0.42]	28.91 [− 34.27, 92.09]	0.16 [0.00, 0.31]	32.23 [0.28, 74.43]	− 0.15 [− 0.62, 0.41]
PD	15	0.54 [0.28, 0.77]	0.71 [0.51, 0.86]	0.17 [− 0.07, 0.41]	44.06 [− 45.40, 133.51]	0.17 [0.01, 0.36]	44.27 [1.77, 124.63]	0.33 [− 0.19, 0.71]
PFF	11	0.52 [0.41, 0.64]	0.64 [0.53, 0.77]	0.13 [− 0.01, 0.26]	26.03 [− 11.30, 63.35]	0.13 [0.06, 0.25]	26.03 [11.65, 58.40]	0.04 [− 0.53, 0.60]
All	82	0.57 [0.35, 0.82]	0.68 [0.52, 0.84]	0.11 [− 0.10, 0.33]	24.48 [− 32.34, 81.30]	0.13 [0.01, 0.30]	26.47 [1.62, 71.34]	0.33 [0.12, 0.51]

Values are either provided as mean and [5%, 95%] quantile or as mean and limit of agreement, if indicated by the limits of agreement (LoA). The first column shows the number of datapoints (n-DPs) used to calculate the statistics.

### Combined evaluation

**Figure 3:**
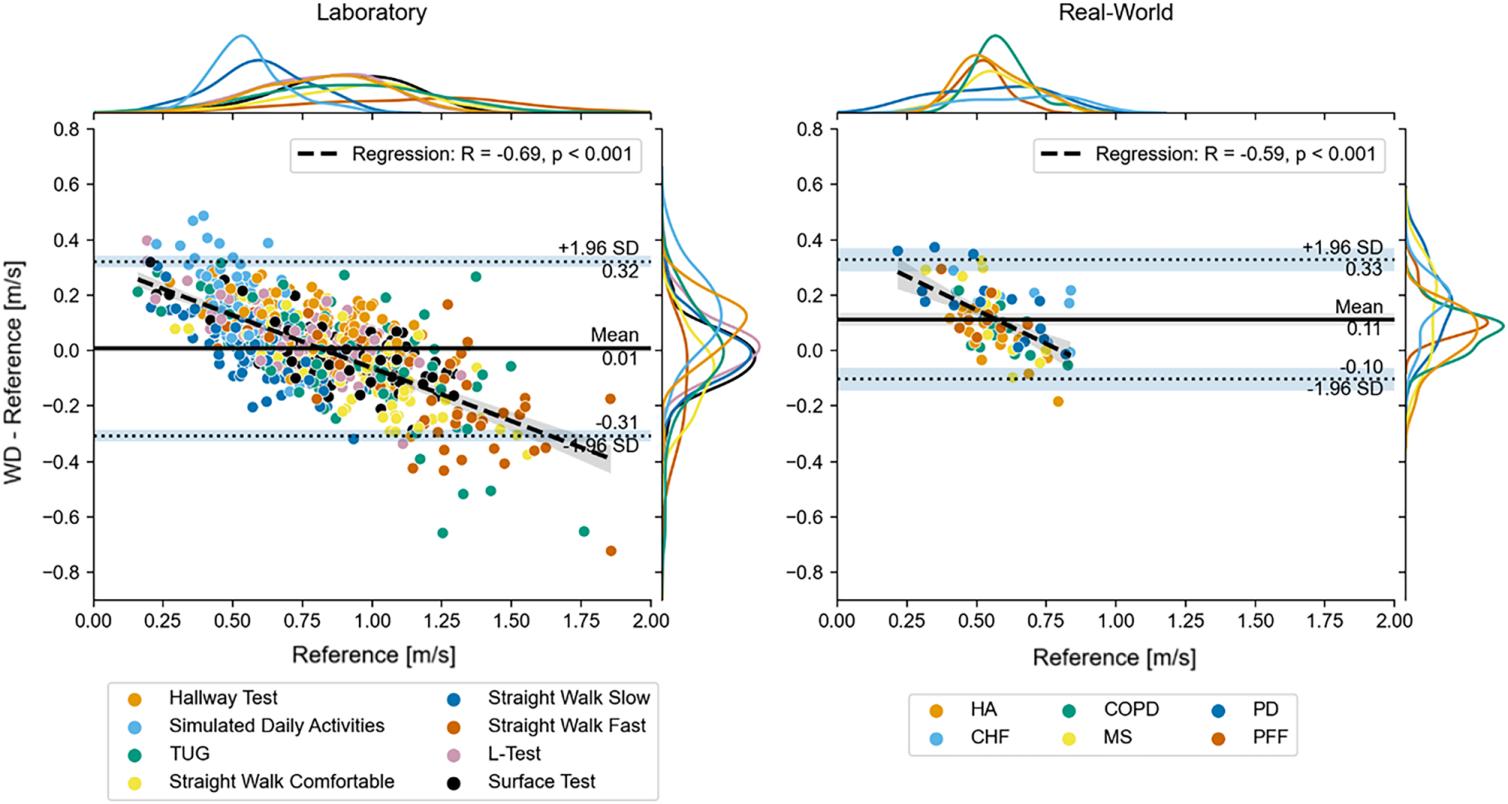
Residual plots for the walking speed combined over all identified WBs. For the laboratory tests the median over all WBs within one motor task is taken (left). For the real-world recording the median over all WBs in the entire real-world assessment is shown (right), where each datapoint represents an individual participant. The margin plots represent the overall speed and error distributions. The margin plots are further grouped by the performed tests for the laboratory and by the cohort for the real-world recordings. The light blue bars around the Limits of Agreement (LOA) (dotted horizontal lines) represent their bootstrapped confidence intervals. The dashed black line represents the result of a linear regression on all datapoints. The grey area around the regression line represents the bootstrapped 95% confidence intervals.

To remove potential bias by focusing only on the true-positive WBs and to mimic actual use of wearable device where reference data may not be available, we performed a second evaluation for which we combined all WBs for a Laboratory test and 2.5 h recording in the real world by taking the median of the calculated DMOs (see Methods). These combined values were then compared between the systems. Results from laboratory data showed a mean error over all tests of 0.01 m/s (MRE = 7.47%) and a MAE of 0.12 m/s (MARE = 17.82%) (Fig. 3, left. Table 4). In contrast, in the real-world we observed a higher mean error over all participants of 0.11 m/s (MRE = 24.48%) and a MAE of 0.13 m/s (MARE = 26.47%) (Fig. 3, right. Table 4). For both environments the errors were higher than those estimated from the analysis on true-positive WBs. The biggest effect was seen on the ICC during the real-world recording which dropped considerably to 0.33 (*poor)* across all cohorts and showed no (PFF: 0.04) or even negative correlation (MS: − 0.15). This is not surprising due to the limited number of datapoints included for this type of analysis (one datapoint per participant).

#### Factors that can influence walking speed validity

##### Influence of the cohort

The MAE based on the true-positive evaluation differed by < 0.05 m/s between cohorts in both laboratory and real-world settings. In the laboratory, the COPD cohort had the lowest MAE (0.06 m/s) followed by HAs (0.08 m/s) (Table 2), whereas the PFF and CHF cohorts had the largest MAE of 0.12 m/s. In the real-world, HAs presented the lowest MAE (0.09 m/s) followed by the PFF cohort (MAE = 0.11 m/s) (Table 2). Walking speed tended to be overestimated for all cohorts apart from CHF, for which walking speed was underestimated by 0.06 m/s in the laboratory and 0.04 m/s in the real-world.

##### Influence of WB duration and walking speed

**Figure 4:**
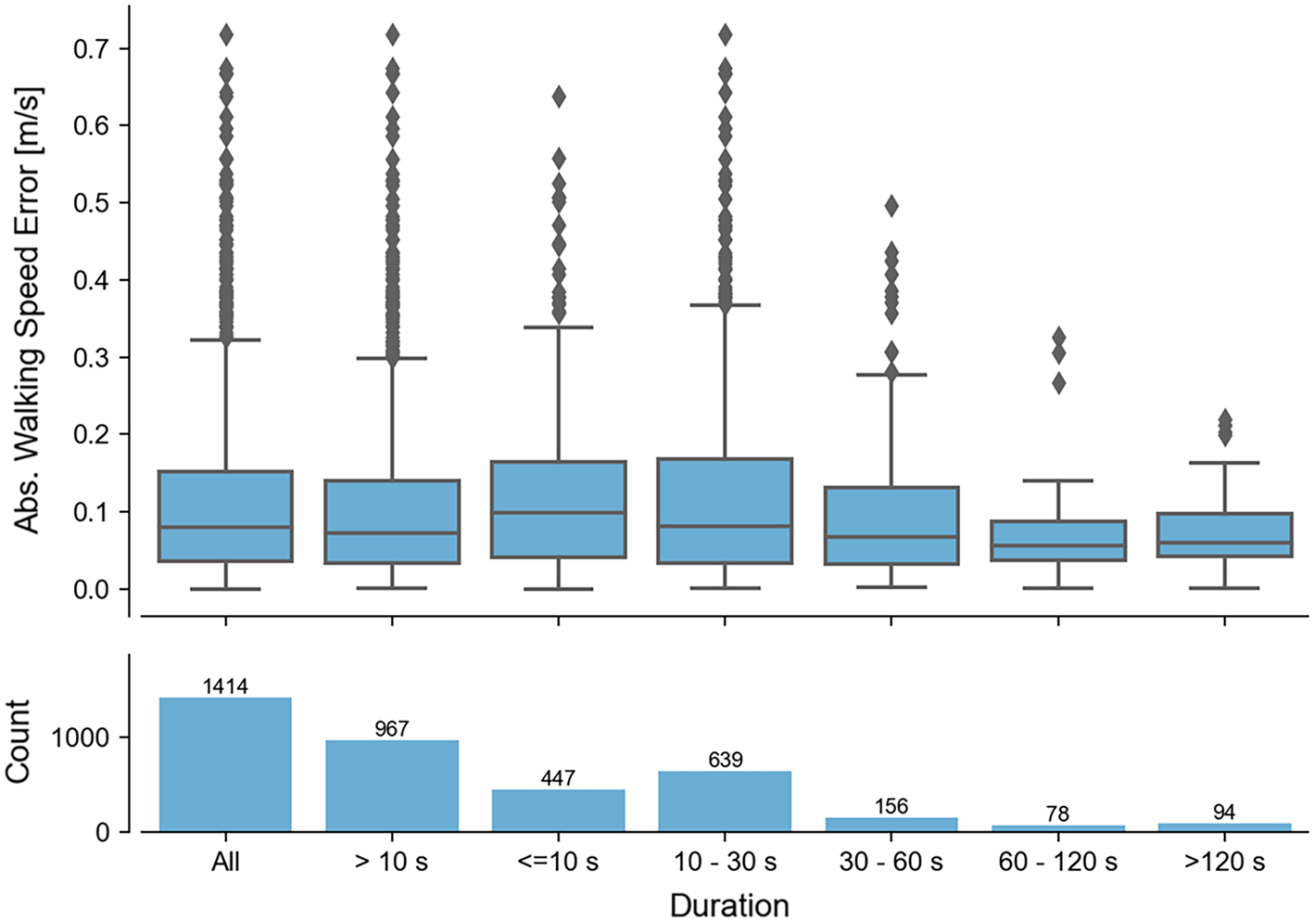
The dependency of the absolute walking speed error of all true-positive WBs from the real-world recording on the WB duration reported by the reference system. In the top, WB errors are grouped by various duration bouts. In the bottom the number of bouts within each duration group is visualized.

**Figure 5:**
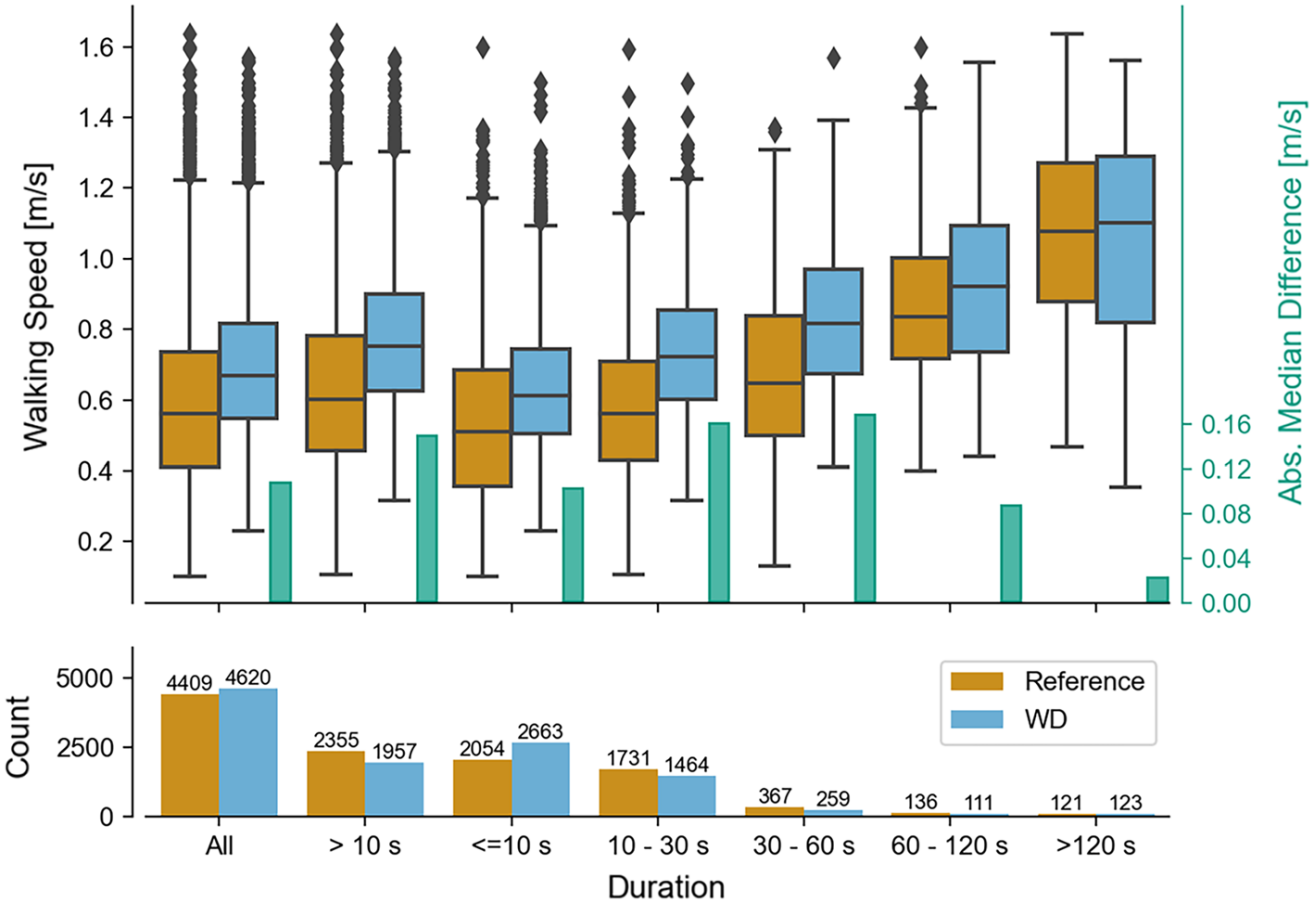
The walking speed estimations from the real-world recording of the reference system and the wearable device, from all WB within the respective duration bouts. The boxplots show the distribution over all WBs. The bars in the upper plot show the absolute difference between the medians of the distributions (see right y-axis). The bottom plot shows the number of WBs in each duration bout.

In the analysis based on the true positive WBs, errors decreased for longer WB durations (Fig. 4). MAE across all cohorts for very short WBs < 10 s ranged between 0.09 and 0.16 m/s, compared to 0.06–0.11 m/s for long WBs (between 60 and 120 s). However, as WB duration increased, the number of available WBs included in the validation analysis decreased as well. When looking at the combined approach (across all detected WBs) (Fig. 5) the trends from the true-positive analysis were confirmed. The MAE for the very short WBs (< 10 s) was lower than for the short WBs (10–30 s), and the number of very short WBs detected by the wearable device was disproportionally higher compared to the reference system. Overall, about two thirds of all WBs were shorter than 30 s. When removing very short WBs (< 10 s) from the calculation of the mean/median errors, the range in error was marginally smaller for some cohorts than the error observed at all WBs for the true positive analysis (improvement < 0.1 m/s). The median of the absolute difference of the combined analysis increased from 0.1 m/s over all WBs to 0.14 m/s for the WBs longer than 10 s.

In both environments, a clear linear negative relationship between the magnitude of the reference walking speed and the measurement errors was observed (Fig. 2). For the slowest WBs (< 0.6 m/s), we observed the largest absolute errors, increasing to 0.8 m/s in real-worlds WBs. Walking speed tended to be overestimated for slow WBs and underestimated for fast WBs. This trend can also be observed in the overall speed distribution of the WBs (Supplementary Fig. 2), which shows a larger number of slow walking bouts and a lower median gait velocity for the reference system compared to the wearable device.

##### Influence of task complexity

**Figure 6:**
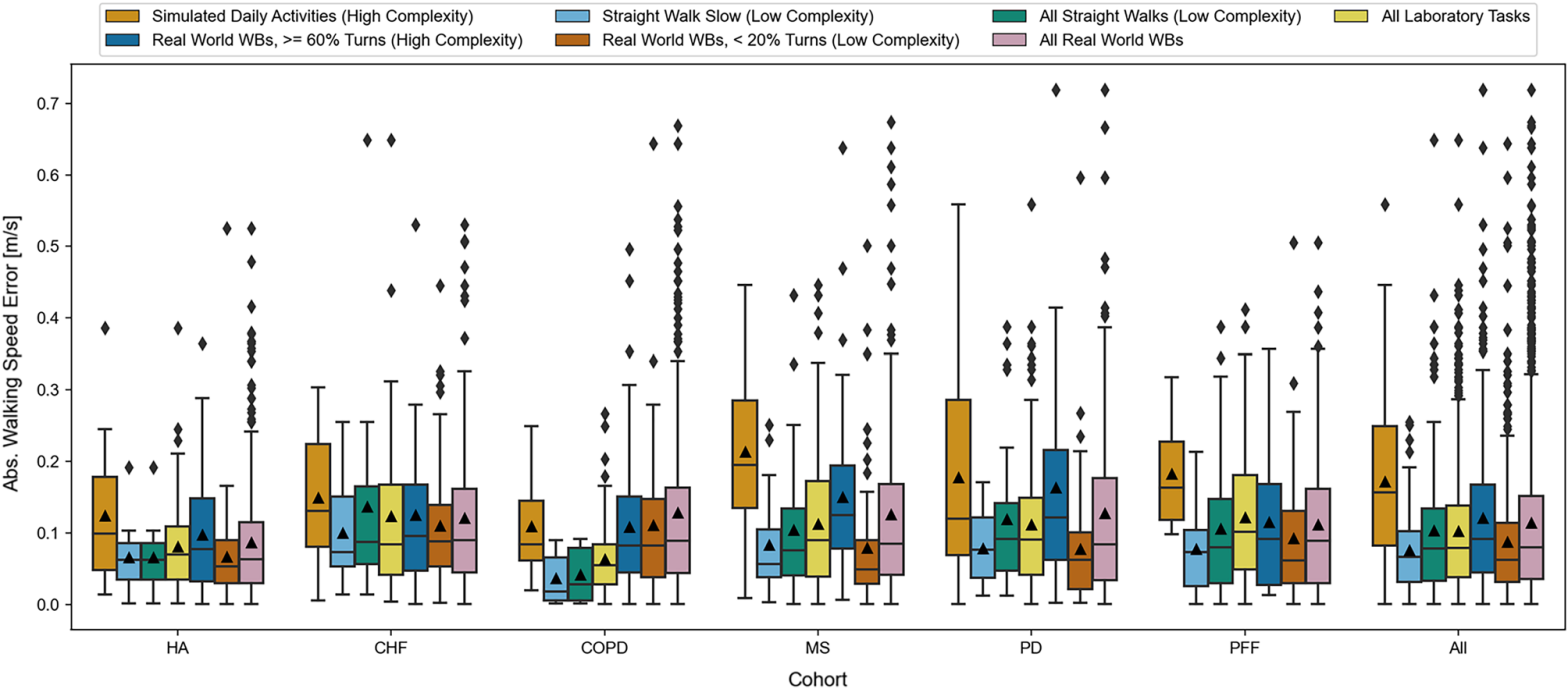
The dependency of the absolute walking speed error on the different defined complexity tasks (see text). The results are split by patient cohort. The “All” group represents the statistics over all WBs independent of the cohort.

As task complexity increased, so did the MAE. For instance, the most complex laboratory gait task (“simulated daily activities”) presented the highest MAE across all cohorts (0.17 m/s) and the least complex task (the slow straight walking test) presented the lowest MAE (0.08 m/s) (Table 5, Fig. 6). Furthermore, the influence of task complexity was cohort dependent. The largest differences between the simple and complex gait tests were observed for the MS, PD, and PFF cohorts (P1 pipeline). In the real-world, for the same cohorts, differences were observed in the errors estimated between the WBs without turns and WBs with turns. For all cohorts, mean error and MAE from real-world assessments were comparable or slightly lower than from simulated daily activities.

**Table 5:** Dependency on complexity for a selection of the gait tasks.

Cohort	Error with LOA (m/s)	Rel. error with LOA (%)	Abs error (m/s)	Rel. abs. error (%)
All straight walks (low complexity)				
HA	− 0.03 [− 0.18, 0.11]	− 5.88 [− 30.92, 19.16]	0.07 [0.01, 0.13]	10.83 [1.47, 24.03]
CHF	− 0.12 [− 0.44, 0.19]	− 11.28 [− 33.35, 10.78]	0.14 [0.03, 0.31]	12.99 [2.90, 29.27]
COPD	− 0.03 [− 0.13, 0.07]	− 3.41 [− 17.78, 10.95]	0.04 [0.00, 0.09]	5.80 [0.38, 14.00]
MS	− 0.02 [− 0.29, 0.25]	0.67 [− 38.85, 40.18]	0.10 [0.02, 0.25]	14.40 [1.31, 37.39]
PD	− 0.04 [− 0.34, 0.26]	0.50 [− 34.73, 35.72]	0.12 [0.01, 0.34]	14.86 [1.64, 33.08]
PFF	− 0.03 [− 0.30, 0.25]	3.38 [− 40.07, 46.82]	0.11 [0.01, 0.32]	15.29 [1.19, 55.27]
All	− 0.04 [− 0.31, 0.23]	− 0.87 [− 37.34, 35.61]	0.10 [0.01, 0.33]	13.66 [1.16, 36.71]
Straight walk slow (low complexity)				
HA	− 0.03 [− 0.18, 0.11]	− 5.88 [− 30.92, 19.16]	0.07 [0.01, 0.13]	10.83 [1.47, 24.03]
CHF	− 0.10 [− 0.26, 0.06]	− 12.64 [− 31.78, 6.50]	0.10 [0.02, 0.22]	13.02 [2.34, 26.89]
COPD	− 0.02 [− 0.11, 0.08]	− 2.89 [− 17.59, 11.82]	0.04 [0.00, 0.09]	5.51 [0.36, 14.06]
MS	0.02 [− 0.18, 0.23]	6.15 [− 38.39, 50.69]	0.08 [0.03, 0.22]	16.58 [4.01, 51.47]
PD	0.01 [− 0.18, 0.19]	4.42 [− 28.15, 37.00]	0.08 [0.01, 0.15]	13.55 [1.72, 35.43]
PFF	0.05 [− 0.12, 0.22]	14.55 [− 33.80, 62.91]	0.08 [0.01, 0.19]	19.00 [1.13, 66.73]
All	0.00 [− 0.18, 0.19]	3.14 [− 36.01, 42.28]	0.08 [0.01, 0.19]	14.14 [1.01, 40.68]
Simulated daily activities (high complexity)				
HA	0.09 [− 0.18, 0.36]	16.54 [− 29.66, 62.73]	0.12 [0.03, 0.30]	20.54 [3.77, 51.48]
CHF	0.13 [− 0.12, 0.38]	29.99 [− 28.01, 88.00]	0.15 [0.03, 0.29]	32.24 [3.41, 64.94]
COPD	0.02 [− 0.25, 0.30]	15.23 [− 48.13, 78.59]	0.11 [0.03, 0.22]	24.87 [4.06, 61.21]
MS	0.19 [− 0.11, 0.49]	37.12 [− 27.99, 102.24]	0.21 [0.06, 0.40]	39.93 [6.36, 96.34]
PD	0.16 [− 0.16, 0.49]	37.65 [− 56.98, 132.29]	0.18 [0.00, 0.36]	39.55 [0.47, 149.25]
PFF	0.18 [0.03, 0.33]	40.29 [− 3.19, 83.78]	0.18 [0.10, 0.30]	40.29 [16.66, 77.12]
All	0.15 [− 0.14, 0.43]	32.39 [− 35.64, 100.43]	0.17 [0.02, 0.37]	35.14 [2.42, 82.42]
All Real World WBs				
HA	0.04 [− 0.18, 0.27]	10.53 [− 35.92, 56.98]	0.09 [0.01, 0.26]	15.45 [0.83, 56.00]
CHF	− 0.04 [− 0.35, 0.27]	1.29 [− 48.12, 50.70]	0.12 [0.01, 0.32]	15.48 [1.24, 53.42]
COPD	0.11 [− 0.16, 0.38]	22.71 [− 43.84, 89.26]	0.13 [0.01, 0.40]	24.88 [1.05, 91.11]
MS	0.09 [− 0.20, 0.39]	19.56 [− 48.42, 87.53]	0.13 [0.01, 0.37]	23.53 [1.70, 80.64]
PD	0.08 [− 0.24, 0.40]	17.79 [− 52.94, 88.53]	0.13 [0.01, 0.38]	22.99 [0.64, 94.50]
PFF	0.04 [− 0.25, 0.32]	10.63 [− 50.73, 71.98]	0.11 [0.01, 0.33]	20.02 [1.24, 73.39]
All	0.06 [− 0.23, 0.35]	14.48 [− 47.17, 76.13]	0.11 [0.01, 0.36]	20.31 [0.95, 76.20]

The results are shown for each cohort, with limits of agreement (LoA). The “All” represents the statistics over all walking bouts (WBs) independent of the cohort.

## Discussion

To our knowledge, this study is the most extensive validation of a complex comprehensive multi-stage analytical pipeline for estimation of walking speed from a single wearable device. Overall, our findings showed good to excellent validation results in the laboratory and moderate to good agreement in the real-world. We demonstrated that validity of walking speed estimation is slightly impacted by several factors including environment (laboratory vs real-world), clinical cohort, gait task complexity and other confounding factors (number of turns, WB duration, WB speed). Our results have strong implications for future research, below we provide our recommendations for future validations and on the use of wearable device-based walking speed in daily life and more broadly, DMOs in general.

### Overall validation results

Overall, laboratory walking speed demonstrated excellent agreement with the reference system, with the ICCs of the true positive WBs ranging from good (0.79) to excellent (0.91) and MAEs ranging from 0.06 to 0.12 m/s across all cohorts. Within the combined evaluation, the ICC of walking speed was slightly lower (0.72–0.82), indicating that only a small difference was introduced by the true positive evaluation. Previous studies conducted across various HAs and various clinical cohorts in laboratory settings have shown lower or comparable results^27,32–34^. However, in comparison to those studies, the pipelines in this study were validated over a wider variety of more complex gait tasks, challenging the estimation of walking speed as the signals are more variable and less cyclic, in comparison to steady-state and straight path gait.

Estimating walking speed in real-world gait assessment poses challenges due to the complexity and non-standardized nature of environments. This difficulty is supported by previous literature, which has found that real-world assessments present a greater challenge for DMO estimation^35,36^.

Despite these challenges, we achieved good results, since agreement was found to be moderate to good (ICCs within true positive WBs ranging between 0.57 and 0.88) and MAE ranged from 0.09 to 0.13 m/s. As regards to the combined real-world WB analysis, the ICCs were lower than the ICCs from true positive WBs. The MAE remained within usable ranges (< = 0.18 m/s), but MARE increased up to 44% primarily due to large relative errors for low gait speeds. In the combined analysis, median average walking speeds for each participant was calculated, which may have increased the impact of individual datapoints with larger errors, as there was only one data point per participant. In some instances, we also observed negative ICCs (MS cohort = − 0.15), which indicates a very poor correlation. Furthermore, this analysis reduced the range in walking speeds, where a larger number of slow WBs were included, which further increased the estimation error.

The WB detection results further show that, dependent upon the cohort, the detected WBs on average only cover between 57 and 72% of the overall walking present in the data. As the pipeline is tuned to provide high specificity, this relatively low sensitivity is expected and this difference in the underlying data distribution partially explained the increased error values for the combined analysis. This demonstrated the bias introduced in the true-positive analysis. Furthermore, the combined approach aggregates real-world data into singular values, which does not reflect the entire distribution of walking speed^37^.

Comparing to one of the few other studies that performed a real-world validation of walking speed, the work by Soltani et al.^38^ validated an algorithm based on a single wrist-worn sensor against a head-mounted Global Navigation Satellite System device, finding low bias [interquartile range (IQR) = − 0.01, 0.00 m/s] and an accuracy expressed by root mean square error [IQR = 0.04, 0.06 m/s]. However, this validation was only performed in 30 HAs (mean age = 37 years)^38^. Given the promising results we report for estimation of walking speed, and the results provided for the individual algorithmic blocks previously reported^26^, we demonstrate that it is possible to use a single wearable device on the lower back for accurate quantification of mobility. However, it must be considered that the performance of algorithms and pipelines are dependent upon a variety of factors that should be taken into consideration during study design, future validation, and data interpretation.

### Recommendation for real-world DMO validation

#### Validation protocol

Across all cohorts we observed larger absolute errors and lower ICCs with walking speed estimated from the real-world in comparison to laboratory assessment, showing the importance of real-world validations to obtain realistic and ecologically valid error estimates of DMOs.

Despite this, our results also show that some real-world challenges can be replicated within laboratory settings, as the errors observed during the simulated-daily activities in the laboratory were in fact higher than in the real-world. In these tasks, participants undertook short WBs containing turns, changes of direction and transitions. Scott et al.^39^ compared the walking speed ranges recorded from the laboratory and 2.5 h protocol that were adopted in the present study and found a diverse profile of walking speed ranges in the laboratory that was representative of the walking speed range observed in the real-world. Future validation studies should take into account an adequate balance between challenging tasks (short WBs, turns and transitions) and long uninterrupted walks in the laboratory protocol to properly replicate the expected error ranges from the real-world.

In general, the expected error ranges were dependent upon task complexity. Most condition specific differences were only prevalent in the real-world, which is consistent with previous research reported in HAs and people with MS and PD^13,18,40^. Our findings motivate the inclusion of complex tasks and simulated daily activities into any future laboratory validation. However, we also recommend inclusion of real-world measurements to capture the true range of gait task complexity performed in daily life as well as a myriad of contextual factors, including the distribution of WB duration and walking speed.

#### Reference system

Utilization of the INDIP system as a reference during both the laboratory-based and real-world protocol proved to be successful in overcoming limitations in accuracy, battery life and usability, all of which are common restrictions of real-world reference systems previously adopted in the literature (e.g., wearable camera and GNSS (global navigation satellite system)^38,41^). Specifically, the INDIP system has been validated, showing excellent agreement (ICC > 0.95) and very low MAEs (simulated daily activities =  ≤ 0.05 m/s) against a stereophotogrammetric system in the same cohorts and laboratory protocol as in the present study^31^. The INDIP system was designed to enable the detection of gait and calculation of parameters based on as few assumptions as possible, particularly concerning the type of walking and the walking environment. Gait event detection relies on pressure insoles that are expected to work independently of the setting, and spatial parameter estimation is based purely on physics-based integration methods that estimate the 3D trajectory of the foot. The INDIP’s performance was evaluated based on a complex experimental protocol specifically designed for mobility assessment. Experiments included selected cohorts of participants with various conditions affecting gait characteristics, performing a complex battery of motor tests designed to produce a heterogeneous and broad range of gait patterns. Results showed overall good/excellent reliability and high repeatability and accuracy for the DMOs analyzed across populations, walking speeds, and WBs. Therefore, the INDIP system is a valuable candidate to collect reference standard data for the analysis of gait in real-world conditions^42,43^. Other existing technologies can be used for obtaining reference data “out-of-the laboratory” (e.g., cameras, markerless systems), but they have intrinsic limitations that make their use inefficient (time consuming data analysis or small volume of data capture), less accurate for stride-by-stride description or not robust to quantify specific gait outcomes (e.g., spatial outcomes).

The INDIP system can be used in both laboratory and real-world settings to enable a concurrent validation of walking speed measurements, as provided in the present study. The recording duration of 2.5 h with the INDIP system enabled recording of a wide range of activities and walking speeds.

#### Data analysis

We adopted two approaches to analyzing walking speed, (i) only considering WBs that were directly matched between the wearable device and reference system (true positive evaluation) and (ii) considering the median value of walking speed across all available data (combined analysis).

The true positive evaluation allowed comparisons to be performed with high granularity on a WB level, allowing better understanding of the circumstances under which the wearable device performs best. However, for the true positive analysis we observed bias with regards to the overall walking speed ranges (Supplementary Figs. 1 and 2), resulting in a non-negligible impact on the real-world results. Therefore, the combined analysis is required to confirm observed error ranges and differences in the results of the two approaches should be considered and discussed. Furthermore, this type of analysis introduced the true-positive threshold as a parameter that influences the results. While we could not find a relevant effect of the selection of this parameter value on the walking speed error (Supplementary Figs. 1 and 2), this might influence other DMOs. As our results indicate, no single type of analysis can provide a definite and full picture of the error ranges. Given the lack of other established approaches to perform real-world comparisons of DMOs with a high granularity, we suggest our framework as a basis for future DMO validation studies.

### Practical recommendations for the use of wearable devices for real-world walking speed measurements

Our results demonstrate that walking speed can be estimated accurately and reliably across a range of environments, cohorts, tasks and contextual factors. Based upon our promising validation results, below we provide our recommendations on the use of wearable device for real-world walking speed measurement.

#### Influence of pipeline

For improved understanding of the error, the impact on individual DMOs within the pipeline should be considered. In our case, given the complexity of the respective algorithms, stride length is expected to have a larger contribution to the observed walking speed error compared to cadence (Supplementary Tables 1 and 2)^26^. This motivates further research in more robust methods for spatial parameter estimation. Furthermore, the wearable device seems to record more shorter WBs than the reference, suggesting that longer continuous bouts of walking were split into multiple shorter WBs. Based on specific investigations of such cases, this was often due to limitations of the initial contact and left–right detection. Under challenging conditions (e.g., turns or stairs), these algorithms could not provide reliable stride information leading to a separation of longer periods of walking into multiple WBs, as no valid stride was detected for multiple seconds. This could be the result of the wearable device being positioned on the lower back, where the reference system was also comprised of feet sensors, thus being more robust to quantify gait events across longer periods. The full pipeline is implemented separately for each system, so the combined estimates of all DMOs needed to meet criteria of a WB leads to heterogeneity between systems. This motivates further research in the detection of initial contacts and their laterality under challenging real-world conditions.

#### Walking bout duration

Real-world walking speed encapsulates a rich dataset of mobility that has been undertaken across various WBs which differ in their length, duration, and context. Each WB reflects a different profile of walking in terms of the number of turns, transitions, and periods of straight walking, which influences walking speed measurement. Therefore, it is not surprising that walking speed estimations were influenced by the WB duration, where very short WBs (< 10 s) presented the largest error. Additionally, the wearable device tends to detect a larger number of shorter WBs than the reference system but fewer medium WBs with intermediate durations (Fig. 5). This suggests that the wearable device tends to fragment gait sequences into smaller segments, possibly attributable to mis-detected initial contacts. We speculate that short WBs predominantly took place within confined indoor spaces such as the home environment. While walking speed captured at this short duration does not reflect steady state gait activities, it could still hold valuable information about balance and functional status (e.g., postural transition, weight-shift, sit-to-stand^44^. Algorithms optimized for straight walking in controlled settings had an increased likelihood of higher absolute errors at very short durations. Based on this, we would recommend using a lower cut-off (WBs > 10 s), to trade-off between the number of removed WBs and still including a minimum threshold of 401 WBs, needed to ensure reliability and validity for real-world gait monitoring in a single cohort^39^.

Moving toward clinical application of wearable devices and walking speed measurement, it is important to consider in which specific real-world context wearable devices can quantify mobility most accurately and reliably. Our findings demonstrated that WBs > 30 s provide the most accurate and reliable measurement. WBs > 30 can be characterized as medium to long in their duration. Walking speed estimated from medium length WBs (between 30 and 60 s), may reflect activities of daily living, such as intermittent periods of shopping or undertaking other errands in public spaces outside the home^18,45^. In contrast, longer WBs (> 60 s), typically capture faster walking speeds that are closer to what is already being measured in the laboratory. Thus, walking speed measured in medium length WBs reflects a balance between capturing activities of daily living, and sufficient periods of straight walking activity that enable the robust quantification of walking speed. However, for certain patients walking continuously for 30 s as our cut-off suggests might already be strenuous. Thus, we would recommend using all (WBs > 10 s) to include a balance between capturing a sufficient number of WBs for patients with a variety of condition severities, whilst ensuring walking speed can still be quantified reliably. Future clinical validation studies with a larger number of participants with severe gait impairments are required to confirm the reported error ranges for specific disease populations and can confirm the influence of WB duration upon the functional insight of mobility provided by walking speed^46^.

#### Walking speed

The influence of the speed at which each WB is completed upon the validity of the walking speed is considered a confounding factor of gait analysis^47^**.** When exploring the average walking speed across all WBs, we found that walking speed in WBs undertaken at slower speeds (< 0.6 m/s) tended to be overestimated by ≤ 0.8 m/s. The wearable device and WBs with faster speeds were underestimated, where moderate walking speeds provided the highest accuracy (Fig. 2). Longer WBs (> 60 s) were completed at faster walking speed in comparison to medium length WBs, which were undertaken at moderate speeds. Further, the overall number of slow WBs appears to be smaller for the wearable device. The speed distribution of only the true-positive WBs exhibits a similar shift in distributions but at overall higher speeds (Supplementary Fig. 2). In conjunction with the presented error values, this suggests that slow WBs are detected correctly, but their speed values are overestimated. Notably, these lower speeds were predominantly observed in short WBs. Consequently, this further justifies our recommendation that medium-length WBs provide the right balance between functional relevance and accuracy. Exploration of the clinical properties of walking speed encapsulated within these WBs, will become a topic of research and further investigated in on-going clinical validation efforts^46^. Furthermore, measurements with cohorts consisting of predominantly slow walkers will likely result in larger error ranges. This is consistent with previous research that also validated algorithms based upon a single lower back sensor^33,48^.

The algorithms used in this study were optimized and developed based on independent datasets to avoid bias. We foresee that future algorithms developed on the TVS dataset, and other similar real-world datasets can improve on the speed dependency observed here.

#### Real-world complexity

Aside from the WB duration, accuracy of walking speed estimation was also dependent upon the complexity of tasks/activities. The influence of complexity was cohort dependent and had the largest influence upon error for the MS, PD and PFF cohorts (estimated from P1 pipeline). We would expect those cohorts to experience more gait impairments than the CHF, COPD and HA cohorts (estimated from the P2 pipeline). However, whether the observed effect is caused by specific gait properties of the respective cohorts or by shortcomings in the selected algorithms, cannot be concluded based on the performed analysis.

Despite the challenges posed by outdoor environments (changes in terrain, weather and traffic negotiation (humans and vehicles))^49,50^**,** outdoor environments capture more prolonged and uninterrupted walks in comparison to indoor environments, such as the household, which represent more confined and cluttered spaces with limited capacity for completing sequences of straight walking. Thus, we would expect error ranges from long uninterrupted outdoor walks are expected to be lower than results from confined indoor environments. Therefore, the combination of gait parameters with further contextual information might help to take this into account during data interpretation.

### Limitations

While this study is one of the largest and most comprehensive validation studies for gait analysis based on wearable devices to date, the analysis of specific subgroup effects would require larger sample sizes. Potential links between the error, the condition severity and other medical comorbidities could not be established. Furthermore, the effect of walking aid use on results has not been assessed in this study. Future studies with more variable condition severity are needed to explore the influence of walking aid usage upon the validity of the analytical pipelines.

The real-world data was limited to 2.5 h for technical reasons. However, we accept that a recording of this length may not be sufficient to capture all the variability and patterns that would be included in multiple days of consecutive assessment. Due to technical issues with the devices, we were unable to assess some participants, which reduced our dataset. Data was also collected during the COVID-19 pandemic which may have impacted on participants’ activity. While the analytical pipeline offers several strengths, its combined implementation does have limitations. As previously stated, the analytical pipelines for the wearable device data have the tendency to split longer WBs into multiple individual WBs. Hence, future research should explore whether this is caused by limitations of the initial contact and Left–Right detections, and how specific real-world contexts may influence walking speed performance. We found that error in walking speed estimation was more dependent upon stride length (spatial) estimation (MARE across all cohorts; laboratory = 14.31% and real-world 20.35%), however cadence (temporal) can be estimated with substantially lower errors (MARE across all cohorts; laboratory = 4.1% and real-world 4.8%) (Supplementary Tables 1 and 2). Therefore, future researchers looking to further improve performance of walking speed estimation, should target optimization of spatial algorithms.

## Conclusion

Through the extensive real-world and laboratory validation across multiple cohorts, this study represents, to the best of our knowledge, the most accurate estimate of the expected error ranges of a lower-back wearable device for estimation of walking speed. The presented state-of-the-art algorithms pipelines could reliably estimate DMOs across a wide range of scenarios, providing a solid foundation for future studies to establish their clinical meaningfulness^46^. While complex setups like camera-based motion capture systems in the laboratory and wearable multi-modal sensor system in real-world scenarios still provide superior performance and might be required for certain types of clinical analysis, we demonstrated the suitability of a single easy-to-use and inexpensive wearable device for movement monitoring across a wide range of clinical indications. This has the potential to make gait related parameters from long-term real world recordings ubiquitously available for clinical decision making. Our results showed that various parameters can influence DMO performance and multi-faceted analysis is crucial for understanding of the capabilities of any DMO pipeline. This motivates the capture of additional context information during real-world measurements to focus analysis on signal areas where high reliability can be expected. Furthermore, we identified clear areas where future algorithm pipelines can still improve, and we believe that the captured dataset will be vital for the development of future algorithms specifically targeting the challenges of unsupervised real-world recordings.

## Methods

### Participants

For the Mobilise-D technical validation study (TVS), participants were recruited from five clinical cohorts (CHF, COPD, MS, PD, and PFF) alongside HA. Participants were recruited at five sites: The Newcastle upon Tyne Hospitals NHS Foundation Trust, UK (Sponsor of the study) and Sheffield Teaching Hospitals NHS Foundation Trust, UK (ethics approval granted by London – Bloomsbury Research Ethics committee, 19/LO/1507); Tel Aviv Sourasky Medical Center, Israel (ethics approval granted by the Helsinki Committee, Tel Aviv Sourasky Medical Center, Tel Aviv, Israel, 0551-19TLV), Robert Bosch Foundation for Medical Research, Germany (ethics approval granted by the ethical committee of the medical faculty of The University of Tübingen, 647/2019BO2), University of Kiel, Germany (ethics approval granted by the ethical committee of the medical faculty of Kiel University, D438/18). Informed consent was provided by all participants to take part in the study and all research was performed in accordance with the Declaration of Helsinki. Inclusion and exclusion criteria are fully described in^24^.

### Protocol

The protocol has been extensively detailed in^24^. Participants were assessed in the laboratory and during a 2.5-h real-world observation. Mobility data was collected with a wearable device (McRoberts Dynaport MM+ , sampling frequency: 100 Hz, triaxial acceleration range: ± 8 g/resolution: 1 mg, triaxial gyroscope range: ± 2000 degrees per second (dps)/ resolution: 70 mdps), secured at the lower back with a Velcro belt. Participants were also asked to wear a multisensor INDIP reference system (sampling frequency: 100 Hz)^24,30^. Specifically, two magneto-IMUs were positioned over the instep and fixed to shoelaces with clips, and a third IMU was attached to the lower back with Velcro. Distance sensors were then positioned asymmetrically with Velcro (one above left ankle and another 3 cm higher on the right leg). Pressure insoles were selected for each participant’s foot size and inserted into the shoe. The INDIP system has been validated in previous studies across a range of conditions and in this TVS cohorts also, showing excellent results and reliability in the qualification of mobility outcomes (MAE laboratory ≤ 0.02 m/s, simulated daily activities = 0.03 to 0.05 m/s), a complete overview of the validation results can be found in^31^. The INDIP and the wearable device were synchronized using their timestamps (± 10 ms). Participants only performed tasks that they felt comfortable and safe to do in both protocols.

#### Laboratory protocol

Participants were asked to complete seven motor tasks with increasing complexity: Straight walking (slow, normal and fast speed), Timed Up and Go, L-Test, Surface Test, Hallway Test and Simulated Daily Activities. Each task was designed to capture and assess various elements associated with real-world walking including a range of walking speeds, incline/steps, surface, path shape, turns and specific motor tasks to simulate typical real-world transitions^24,39^.

#### Real-world protocol

Participants were assessed for up to 2.5 h in the real-world, as they went about their normal activities unsupervised (home/work/community/outdoor). The duration of the observation has been established as a trade-off between experimental, clinical, and technical requirements. To capture the largest possible range of activities during this assessment, participants were guided by the following list of activities: if relevant for their chosen environment, rise from a chair and walk to another room; walk to the kitchen and make a drink; walk up and down a set of stairs (if possible); walk outdoors (if possible, for a minimum of 2 min); if walking outside, walk up and down an inclined path. We did not provide supervision or structure on how these tasks should be completed to the participants^24^.

### Calculation of walking speed

The evaluation of walking speed requires the combination of various algorithmic steps, including the identification of gait sequences and of initial contacts, estimation of DMOs, i.e., cadence and stride length. Selection of the top-ranked algorithms to detect gait-sequences, estimate initial contact events, cadence and stride length within identified gait-sequences was determined in our previous work^26^ (Fig. 1). The best performing algorithm was then used to estimate walking speed using the outputs of the stride length and cadence algorithms using Eq. (1):

1 $$Walking\;speed\left[ {m/s} \right] = \left( {cadence\left[ {step/min} \right]/\left( {2*60} \right)} \right)*stride\, length\left[ m \right]$$

**Figure 7:**
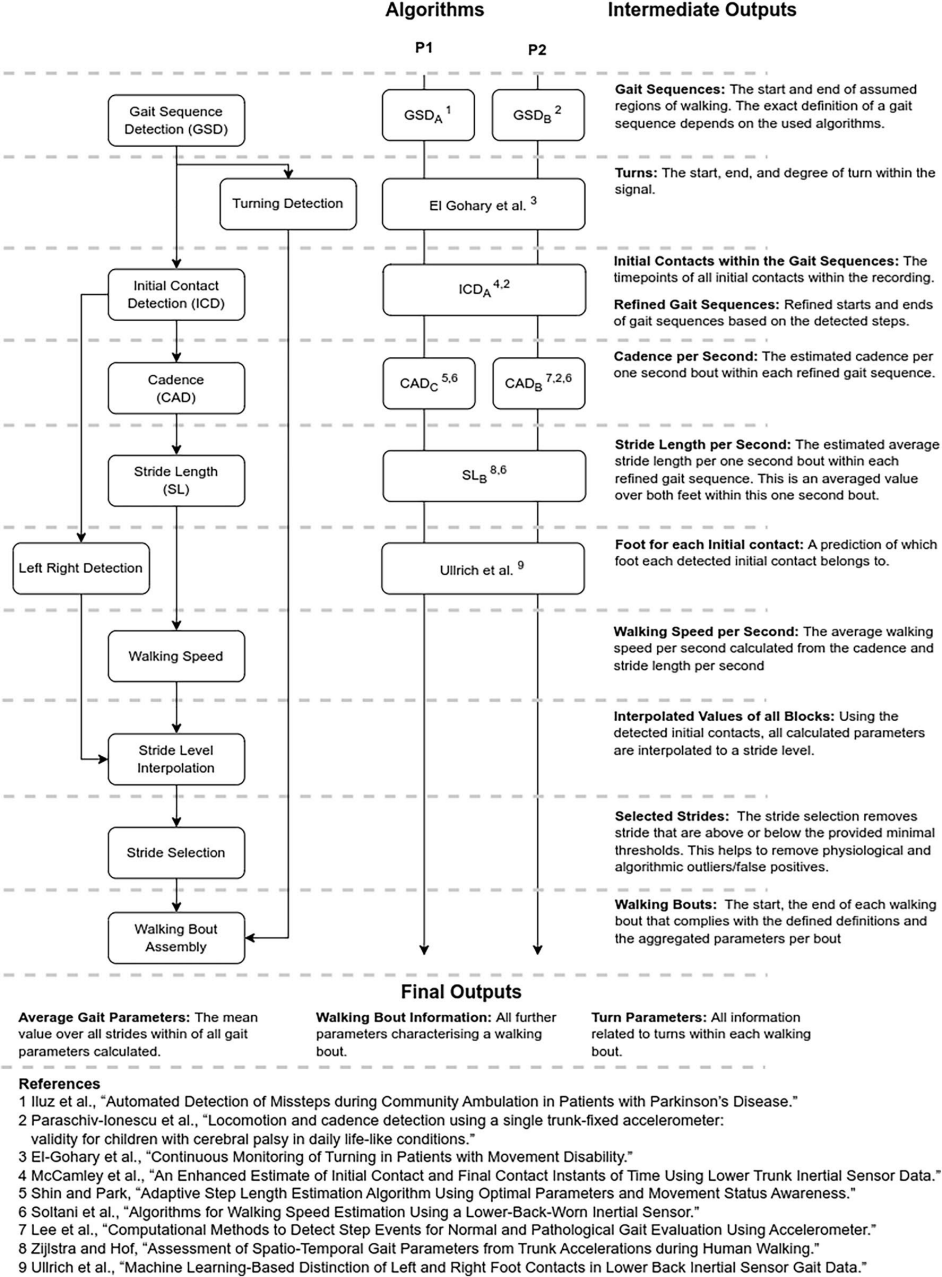
Overview over the different algorithmic steps of the analytical pipeline with short explanations of the intermediate and final outputs of each of the algorithmic blocks; gait sequence detection (GSD), initial contact detection (ICD), cadence estimation (CAD) and stride length estimation (SL). The algorithm column indicates the used algorithms for the two pipelines P1 (HA, COPD, CHF). (MS, PD, PFF) and P2 (MS, PD, PFF) Short citations for the algorithms are provided below the figure. For more details see Table 1 in^26^.

Two independent analytical pipelines (P1 and P2) were identified in this process due to differences in algorithm selection for gait-sequence detection and cadence for the different conditions included in the study^26^. P1 provides the optimal combination of algorithms selected for HA, COPD, and CHF conditions, and P2 provides the optimal combination for PD, MS, and PFF (Fig. 7).

Two additional algorithms were added to both gait analysis pipelines: turn detection algorithm^51^, and a customized algorithm to detect the laterality (left or right step) of each IC^52^. Laterality was used to interpolate the cadence, stride length, and walking speed parameters (provided as per-second values by the algorithms) to stride-level values (*stride interpolation*).

DMOs were evaluated on a stride level, conforming to consensus agreed definitions^53^ for WBs. Accordingly, a WB was defined as a continuous sequence containing at least two consecutive strides of both feet (e.g., R–L–R–L–R–L or L–R–L–R–L–R, being R/L the right/left foot making contact with the ground); consecutive WBs were defined if a break greater than 3 s was identified between them; and, for a stride to be included in a given WB it had to have a duration between 0.2 and 3.0 s and a stride length > 0.15 m^54^. WBs compliant with this definition were generated by first filtering the list of identified strides based on the stride level definition (*stride selection*) and then grouped into final WBs based on breaks within the stride sequence (*walking bout assembly*). The same definitions were also used to define the WBs for the reference system. For both systems final DMOs were calculated as the average value over all strides within a WB.

### Validation of walking speed

All comparisons between the wearable device and the reference system were performed based on the average walking speed within each identified WB. In addition, comparison results for cadence and stride length can be found in Supplementary Tables 1 and 2. Further, we evaluated the performance of the WB detection on the 2.5-h real-world assessment to provide additional context for the error parameters. For this we calculated the accuracy, sensitivity, specificity and positive predictive value, by comparing the regions of the WBs detected by the wearable device with the WBs reported by the reference system on a sample-by-sample basis following the same approach used for evaluation of the gait sequence detection (GSD) methods in^26^. Real-world recordings also provide new challenges during data analysis. WBs detected by the wearable device, and the reference system might not match up, thus direct comparison of individual strides or WBs is not possible. One straight-forward approach is to average DMOs across all WBs before comparison. However, this reduced granularity makes it difficult to fully understand under which circumstances a wearable device works well and can “mask” the bias or error (e.g., over or underestimation under specific circumstances) that only considering a single WB could be identified. We proposed a new approach for these types of data analysis, by splitting the analysis into a detailed comparison of only WBs that were identified in both systems (True positive WBs) and a traditional analysis of all data combined (Combined WBs).

1. *True Positive Evaluation* Novel method of analysis, which directly compares the performance of the DMOs on only the WBs that were detected in both systems (true positives). This allows for the calculation of traditional of comparison metrics (e.g., interclass correlation and Bland–Altman plots), that require a direct comparison of individual measurement points. WBs were included in the true positive analysis, if there was an overlap of more than 80% between the two systems (details about the selection of this threshold can be found in Supplementary Fig. 1). The threshold of 80% was selected as a trade-off to allow us: (i) to consider as much as possible a like-for-like comparison between selected WBs (INDIP vs. wearable device), and at the same time (ii) to include the minimum number of walking bouts to ensure sufficient statistical power for the analyses (i.e., at least 101 walking bouts for each cohort). This target was based upon the number of walking bouts rather than a percentage of total walking bouts that would allow us to meet criteria established by statistical experts for robust statistical analysis after sample-size re-evaluation (total walking bouts number > 101 corresponding to ICC > 0.7 and a CI = 0.2).
2. *Combined Evaluation* Traditional method of analysis, where we calculated the median walking speed from all identified WBs within each laboratory task (resulting in one value per gait task per participant) or within the 2.5 h real-world assessment per participant (resulting in one value per participant) and compared these combined values between the systems. This comparison is free of potential biases introduced by the selection of only the true-positive WB and reflects how DMOs will typically be evaluated in a research or clinical setting or when reference data may not be available.

### Factors that can influence walking speed validity

A range of factors can influence walking speed, and this may impact the algorithm performance and validity of the results. We investigated the possible sources of confounding such as: the cohort, environment (laboratory vs real-world), task complexity, walking speed and walking bout duration, and participant performance upon walking speed validity. All comparisons (unless otherwise stated) are performed using WBs identified as true positive (true positive evaluation).

#### Influence of the cohort and environment

We compared errors in the estimation of walking speed between each of the different clinical cohorts included in the study, alongside differences between laboratory and real-world environments.

#### Influence of gait task complexity

Real world walking contains complex gait sequences, which are comprised of short steps, frequent turns, or obstacle negotiation where individuals often multitask during walking. Thus, gait patterns observed in the real-world are not comparable with the straight walking tasks undertaken in controlled environments, even if we account for differences in WB duration and walking speed. To assess the effect of gait-task complexity we compared validation results of walking speed estimated from the (i) simulated daily activities (high complexity), (ii) slow straight walking (low complexity), (iii) all straight walking tasks (low complexity), and (iv) all laboratory tests with the validation results of walking speed estimated from real-world walking. We further subdivided real world walking based on the percentage of a WB that was assessed to be a turn. Based on this we defined the following levels of gait complexity: (v) “simple” straight gait (< 20% covered by turns) and (vi) “complex gait” (> = 60% covered by turns).

#### Influence of walking speed and walking bout duration

Given the impact of real-world WB durations and speeds^44^ on the adopted biomechanical strategies^55^, we analyzed their influence on the validity of the walking speed. For this, we assessed whether validity of walking speed estimation differed within specific WB durations bins (< 10 s, > 10 s, 10–30 s, 30–60 s, > 60 s and > 120 s). This was first performed for all true positive WBs comparing their errors across each WB threshold, and subsequently repeated for the combined analysis, by calculating the median walking speed for each participant within the respective speed bout and comparing the median values between the reference system and the wearable device. All these analyses permitted the validation of the quantification of walking speed across different walking strategies.

### Validation measures

For all types of evaluations (all available WBs/aggregated values or on the respective subgroups), we calculated various statistical/comparison measures to quantify the walking speed estimation error for the sensitivity analysis:

- Intraclass Coefficient (ICC_(2,1)_)^56^ was calculated to assess the association between the DMOs of the two systems. Based on ICC estimates, values < 0.5, between 0.5 and 0.75, between 0.75 and 0.9, and > 0.90 were deemed to be indicative of poor, moderate, good, and excellent reliability, respectively^57^.
- Absolute agreement was assessed by quantifying (i) the accuracy/mean absolute error (MAE), (ii) bias/mean error and (iii) precision/limits of agreements (LoA)^58^ between walking speed estimates of both systems.
- Mean relative errors (MRE) and mean absolute relative error (MARE) were estimated as the ratio between the (absolute) errors per WB and the corresponding estimates from the reference system, expressed as a percentage.

## Supplementary Information

Supplementary Information.

## Data Availability

Representative data from the dataset presented in this study can be found on Zenodo: https://doi.org/10.5281/zenodo.7547125. The complete dataset of the Mobilise-D Technical Validation study will be made publicly available in the future.
