# Author Correction: Mobilise-D insights to estimate real-world walking speed in multiple conditions with a wearable device

**DOI:** 10.1038/s41598-024-79454-4

**Published:** 2024-11-21

**Authors:** Cameron Kirk, Arne Küderle, M. Encarna Micó-Amigo, Tecla Bonci, Anisoara Paraschiv-Ionescu, Martin Ullrich, Abolfazl Soltani, Eran Gazit, Francesca Salis, Lisa Alcock, Kamiar Aminian, Clemens Becker, Stefano Bertuletti, Philip Brown, Ellen Buckley, Alma Cantu, Anne-Elie Carsin, Marco Caruso, Brian Caulfield, Andrea Cereatti, Lorenzo Chiari, Ilaria D’Ascanio, Judith Garcia-Aymerich, Clint Hansen, Jeffrey M. Hausdorff, Hugo Hiden, Emily Hume, Alison Keogh, Felix Kluge, Sarah Koch, Walter Maetzler, Dimitrios Megaritis, Arne Mueller, Martijn Niessen, Luca Palmerini, Lars Schwickert, Kirsty Scott, Basil Sharrack, Henrik Sillén, David Singleton, Beatrix Vereijken, Ioannis Vogiatzis, Alison J. Yarnall, Lynn Rochester, Claudia Mazzà, Bjoern M. Eskofier, Silvia Del Din, Mobilise-D consortium, Francesca Bottin, Lorenzo Chiari, Cristina Curreli, Ilaria D’Ascanio, Giorgio Davico, Roberta De Michele, Giuliano Galimberti, Luca Palmerini, Saverio Ranciati, Luca Reggi, Marco Viceconti, Lucia D’Apote, Jules Desmond, Megan Doyle, Mary Elliot-Davey, Gilles Gnacadja, Anja Kassner, Beat Knusel, Monika Pocrzepa, Nicolas Pourbaix, Hoi-Shen Radcliffe, Lening Shen, Jennifer Simon, Jesper Havsol, Diana Jarretta, Magnus Jornten-karlsson, Pierre Mugnier, Solange Corriol Rohou, Gabriela Saraiva, Henrik Sillén, Michael Boettger, Igor Knezevic, Frank Kramer, Paolo Piraino, Hubert Trübel, Hajar Ahachad, Hubert Blain, Sylvie Broussous, Francois Canovas, Florent Cerret, Louis Dagneaux, Valerie Driss, Florence Galtier, Charlote Kaan, Stephanie Miot, Eva Murauer, Anne-Sophie Vérissimo, Daniela Berg, Kirsten Emmert, Clint Hansen, Hanna Hildesheim, Jennifer Kudelka, Walter Maetzler, Corina Maetzler, Christian Schlenstedt, Valdo Arnera, Karin Beckstrom, Patrick Folaron, Antonia Gizdic, Fay Horak, Skender Imeri, Stefanie Krieger, Narcis Nica, Natalia Pletneva, Stephen Raymond, Donna Reed, Ara Sekaram, Kristen Sowalsky, Kamiar Aminian, Anisoara Ionescu, Abolfazl Soltani, Bjoern Eskofier, Felix Kluge, Arne Küderle, Martin Ullrich, Victoria Alcaraz Serrano, Magda Bosch de Basea, Joren Buekers, Gabriela Cardenas, Anne-Elie Carsin, Ines Cobo, Anna Delgado Llobet, Laura Delgado Ortiz, Mariona Font Garcia, Judith Garcia Aymerich, Elena Gimeno-Santos, Alicia Jose, Sarah Koch, Ashar Ahmad, Marcel Froehlich, Gilyana Borlikova, Marie-Sidonie Edieux, Ronan Fox, Bill Holt, Kellee Howard, Sean Kelly, Sheila Kelly, Ruth Lalor, Alexandre Malouvier, Kusuma Manavalli Ramanna, Marie Mc Carthy, Gerard Quinn, Isaac Rodriguez Chavez, Peter Schueler, Michal Skackov, Barbara Skerrit, Sara Buttery, Nicholas Hopkinson, Alexis Perkins, Keir Philip, Mike Polkey, Parris Williams, Michael Jackson, David Wenn, Sofie Breuls, Heleen Demeyer, Nitesh Ghosh, Pieter Ginis, Lies Glorie, Valerie Haerens, Lova Hulst, Femke Hulzinga, Wim Janssenns, Alice Nieuwboer, Thierry Troosters, Tim Vanhoutte, Myriam Witvrouw, Marieke Wuyts, Luca Cornelisse, Jordi Evers, Siete Frouws, Neall Mouthaan, Martijn Niessen, Laura Siepman, Aida Aydemir, Yann Hyvert, Martin Aursand Berge, Mara Diaconu, Monika Engdal, Karoline Blix Grønvik, Jorunn Helbostad, Lars Gunnar Johnsen, Anna Marcuzzi, Ingalill Midtsand, Mari Odden, Ingvild Saltvedt, Erika Skaslien, Kristin Taraldsen, Beatrix Vereijken, Ola Bunte, Wim Dartee, Gul Erdemli, Olivier Grenet, Tilo Hache, Sam Hariry, Sabina Hernandez Penna, Felix Kluge, Jacek Lukawy, Suzanne Maahs, Ram Miller, Arne Mueller, Jens Praestgaard, Ronenn Roubenoff, Sandra Schluechter, Leen van Steenbergen, Xuemei Cai, Charmaine Demanuele, Charmaine Demanuele, Mariana Gameiro, Di Junrui, Isik Karahanoglu, Joe Mather, Dimitrios Psaltos, Emma Stokes, Anil Tarachandani, Hao Zhang, Anne-Marie Kirsten, Kirsten Paash, Martina Russ, Henrik Watz, Ines Zimmermann, Clemens Becker, Niki Brenner, Christoph Endress, Martha Gierka, Clarissa Huber, Simon Jaeger, Carl-Philipp Jansen, Bernd Kinner, Jochen Klenk, Elena Litz, Elena Litz, Stefanie Mikolaizak, Kilian Rapp, Matthias Schwab, Lars Schwickert, Erkin Uysal, Martin Wohlrab, Vanessa Zoller, Nadir Ammour, Stephanie Bascle, Fabrice Bonche, Manon Cariou, Matthieu Jouannin, Mike Chambers, Antonella Ciucchiuini, Ariel Dowling, Emilio Merlo-Pich, Max Tolkoff, Lucy Fry, Mark Gordon, Pippa Loupe, Michal Melamed, Michael Reich, Sara Shnider, Marina Brozgol, David Buzaglo, Pablo Cornejo Thumm, Eran Gazit, Nir Giladi, Jeff Hausdorff, Talia Herman, Inbar Hillel, Anat Mirelman, Ayala Saban, Shahar Yehezkyahu, Nikolaos Chynkiamis, Stefano Bertuletti, Marco Caruso, AndreaCereatti, Andrea Manca, Francesca Salis, Valeria Bonanno, Giampaolo Brichetto, Gloria Dalla Costa, Comi Giancarlo, Letizia Leocani, Allia Mahajneh, Matteo Martinis, Mariaemma Rodegher, Andrea Tacchino, Mauro Zaffaroni, Mauro Zaffaroni, Gilbert Buesching, Anja Frei, Katharina Hackl, Melanie Keller, Marion Maggi-Beba, Ashley Polhemus, Milo Puhan, Thomas Riegler, Thomas Sigrist, Sabine Spielmanns, Marc Spielmanns, Valerie Zumbrunnen, Stafanie Dettmer, Heiko Gassner, Teresa Greinwalder, Konstantin Huhn, Jelena Jukic, Jochen Klucken, Franz Marxreiter, Florian Nickel, Martin Regensburger, Veit Rothhammer, Sarah Seifferth, Sabine Stallforth, Tanja Stirnweiß, Andrea Weitzenfelder, Juergen Winkler, Antonio Bevilaqua, Brian Caulfield, Cathy Goulding, Georgiana Ifrim, Tahar Kechadi, Alison Keogh, Brian Mac Namee, Milu Philip, David Singleton, Lisa Alcock, Graham Armitage, Jaume Bacardit, Harry Bailey, Phil Brown, Alma Cantu, Laura Cordova-Rivera, Silvia Del Din, Brook Galna, Ann Gibson, Ashley Hart, Hugo Hiden, Chloe Hinchliffe, Sara Johansson Fernstad, Cameron Kirk, Ellen Lirani-Silva, Encarna Micó Amigo, Isabel Neatrour, Emma Packer, Annette Pantall, Jian Qing Shi, Lynn Rochester, Emily Hume, Dimitrios Megaritis, Ioannis Vogiatzis, Sarah Birchall, Tecla Bonci, Gavin Brittain, Ellen Buckley, Fabio Ciravegna, Sooji Han, Liam Haslam, Neil Ireson, Azza Ishmail, Mahjabin Islam, Vita Lanfranchi, Michael Long, Claudia Mazzà, Jessica McNeil, Shagun Misraq, Sarah Moll, Ahmed Mubarak-Mohamed, Siva Nair, David Paling, Shivani Patel, Dibya Pattanaik, Daisy Priest, Alex Radford, Kirsty Scott, Basil Sharrack, Lubos Vaci, Linda Van Gelder

**Affiliations:** 1 Translational and Clinical Research Institute, Faculty of Medical Sciences, Newcastle University, The Catalyst 3 Science Square, Room 3.27, Newcastle Upon Tyne, NE4 5TG UK; 2 Machine Learning and Data Analytics Lab, Department Artificial Intelligence in Biomedical Engineering, Friedrich-Alexander-Universität Erlangen-Nürnberg, Erlangen, Germany; 3 Department of Mechanical Engineering and Insigneo Institute for in Silico Medicine, The University of Sheffield, Sheffield, UK; 4 Laboratory of Movement Analysis and Measurement, Ecole Polytechnique Federale de Lausanne, Lausanne, Switzerland; 5 Center for the Study of Movement, Cognition and Mobility, Neurological Institute, Tel Aviv Sourasky Medical Center, Tel Aviv, Israel; 6 Department of Biomedical Sciences, University of Sassari, Sassari, Italy; 7 National Institute for Health and Care Research (NIHR) Newcastle Biomedical Research Centre (BRC), Newcastle University and the Newcastle Upon Tyne Hospitals NHS Foundation Trust, Newcastle Upon Tyne, UK; 8 Robert Bosch Gesellschaft für Medizinische Forschung, Stuttgart, Germany; 9 Department of Electronics and Telecommunications, Politecnico di Torino, Turin, Italy; 10 The Newcastle Upon Tyne Hospitals NHS Foundation Trust, Newcastle Upon Tyne, UK; 11 School of Computing, Newcastle University, Newcastle Upon Tyne, UK; 12 Barcelona Institute for Global Health (ISGlobal), Barcelona, Spain; 13 Universitat Pompeu Fabra, Barcelona, Catalonia Spain; 14 CIBER Epidemiología y Salud Pública (CIBERESP), Madrid, Spain; 15 Insight Centre for Data Analytics, University College Dublin, Dublin, Ireland; 16 School of Public Health, Physiotherapy and Sports Science, University College Dublin, Dublin, Ireland; 17 Department of Electrical, Electronic and Information Engineering «Guglielmo Marconi», University of Bologna, Bologna, Italy; 18 Health Sciences and Technologies—Interdepartmental Center for Industrial Research (CIRI-SDV), University of Bologna, Bologna, Italy; 19 Department of Neurology, University Medical Center Schleswig-Holstein Campus Kiel, Kiel, Germany; 20 Department of Physical Therapy, Sagol School of Neuroscience, Sackler Faculty of Medicine, Tel Aviv University, Tel Aviv, Israel; 21 Rush Alzheimer’s Disease Center and Department of Orthopaedic Surgery, Rush University Medical Center, Chicago, IL USA; 22 Department of Sport, Exercise and Rehabilitation, Northumbria University Newcastle, Newcastle Upon Tyne, UK; 23 Novartis Institutes of Biomedical Research, Novartis Pharma AG, Basel, Switzerland; 24 McRoberts BV, The Hague, The Netherlands; 25 Department of Neuroscience and Sheffield NIHR Translational Neuroscience BRC, Sheffield Teaching Hospitals NHS Foundation Trust, Sheffield, UK; 26 Digital Health R&D, AstraZeneca, Sweden; 27 Department of Neuromedicine and Movement Science, Norwegian University of Science and Technology, Trondheim, Norway; 28 Alma Mater Studiorum - Università di Bologna, Bologna, Italy; 29 Amgen, Thousand Oaks, CA USA; 30 AstraZeneca AB, Gothenburg, Sweden; 31 Bayer Aktiengesellschaft, Leverkusen, Germany; 32 Centre Hospitalier Universitaire de Montpellier, Montpellier, France; 33 Christian-Albrechts-Universität, Kiel, Germany; 34 Clario, Philadelphia, USA; 35 Ecole Polytechnique Federale de Lausanne, Lausanne, Switzerland; 36 Friedrich-Alexander-Universitaet Erlangen-Nuernberg, Erlangen, Germany; 37 Fundacion Privada Instituto de Salud Global, Barcelona, Spain; 38 Gruenenthal GMBH, Aachen, Germany; 39 ICON Clinical Research Limited, Dublin, Ireland; 40 Imperial College of Science Technology and Medicine, London, UK; 41 Ixscient Ltd, London, UK; 42 Katholieke Universiteit Leuven, Leuven, Belgium; 43 McRoberts B.V., The Hague, The Netherlands; 44 Merck KGaA, Darmstadt, Germany; 45 Norges Teknisk-Naturvitenskapelige Universitet, Trondheim, Norway; 46 Novartis Pharma AG, Basel, Switzerland; 47 Pfizer Limited, Tadworth, UK; 48 Pneumologisches Forschungsinstitut an der LungenClinic Grosshansdorf GmbH, Großhansdorf, Germany; 49 Robert Bosch Gesellschaft Fur Medizinische Forschung MBH, Stuttgart, Germany; 50 Sanofi Aventis Recherche et Developpement, Chilly-Mazarin, France; 51 Takeda, Tokyo, Japan; 52 Teva Pharmaceutical Industries Ltd, Tel Aviv-Yafo, Israel; 53 The Foundation for Medical Research Infrastructural Development and Health Services, Tel Aviv-Yafo, Israel; 54 Thorax Foundation, Athens, Greece; 55 Università Degli Studi di Sassari, Sassari, Italy; 56 Università Vita-Salute San Raffaele, Milan, Italy; 57 Universitat Zurich, Zurich, Switzerland; 58 Universitatsklinikum Erlangen, Erlangen, Germany; 59 University College Dublin, Dublin, Ireland; 60 University of Newcastle, Newcastle Upon Tyne, UK; 61 University of Northumbria, Newcastle Upon Tyne, UK; 62 University of Sheffield, Sheffield, UK

Correction to: *Scientific Reports* 10.1038/s41598-024-51766-5, published online 19 January 2024

The original version of this Article contained an error in Figure 7 where an incorrect reference was cited for one of the recommended algorithms for Gait Speed Detection (GSD). The original Figure 7 and accompanying legend appear below.

**Figure 7:**
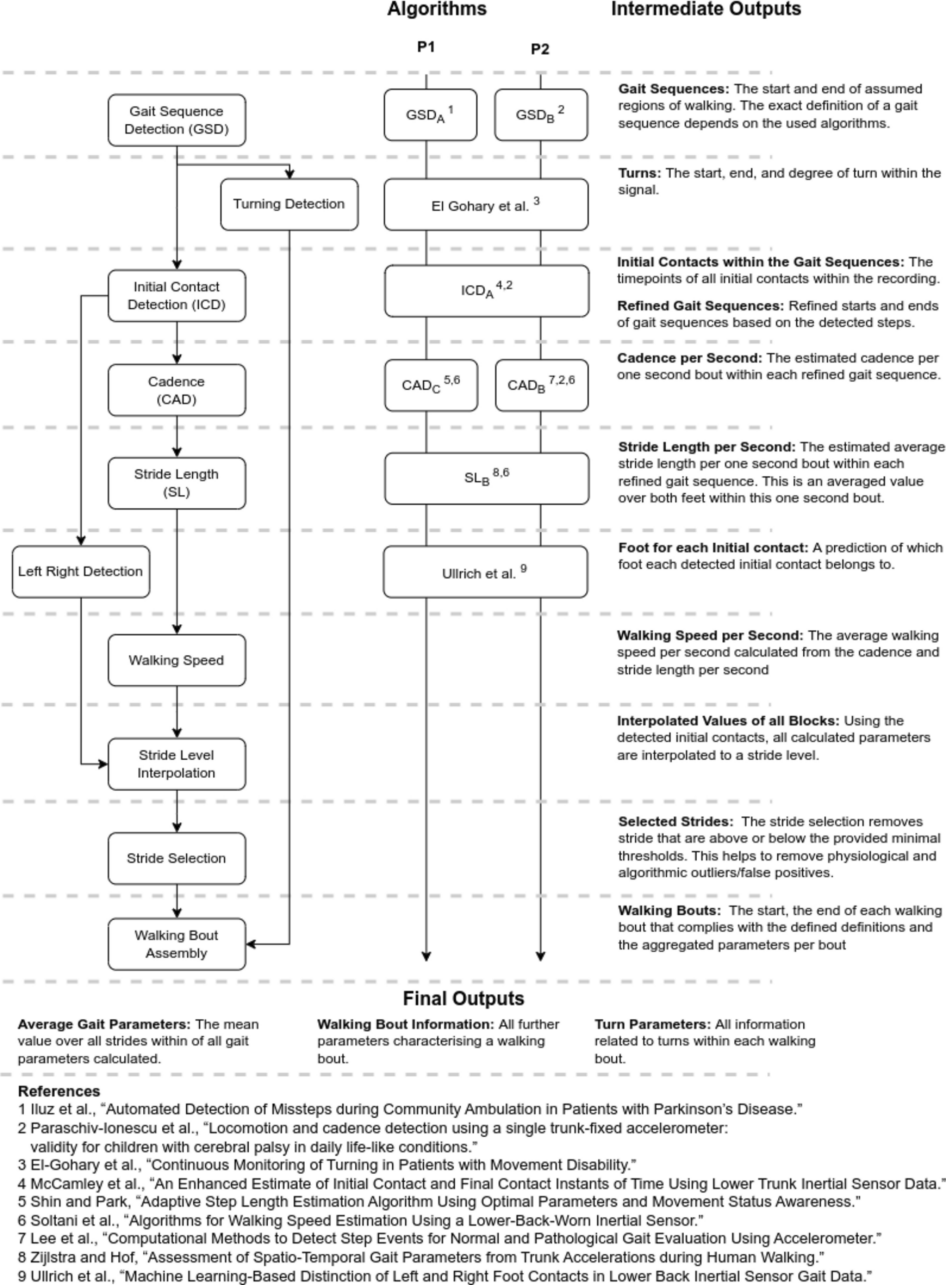
Overview over the diferent algorithmic steps of the analytical pipeline with short explanations of the intermediate and fnal outputs of each of the algorithmic blocks; gait sequence detection (GSD), initial contact detection (ICD), cadence estimation (CAD) and stride length estimation (SL). Te algorithm column indicates the used algorithms for the two pipelines P1 (HA, COPD, CHF). (MS, PD, PFF) and P2 (MS, PD, PFF) Short citations for the algorithms are provided below the fgure. For more details see Table 1 in^26^.

In addition, the Supplementary Information 1 file published with this Article contained errors in Tables 1 and 2. The Intraclass Correlation Coefficients (ICCs) for walking speed were incorrectly reported instead of the correct ICC values for stride length and cadence.

The original Supplementary Information 1 file is provided below.

The original Article and the Supplementary Information 1 file that accompanies the original Article have been corrected.

## Supplementary Information

Supplementary Information.

