# Supplementary material for: Mobilise-D insights to estimate real-world walking speed in multiple conditions with a wearable device
Source: Sci Rep. 2024 Jan 19;14:1754. doi: 10.1038/s41598-024-51766-5 (PMC10799009; doi:10.1038/s41598-024-51766-5)
Supplement: Supplementary file 1 — Supplementary Information. [file 41598_2024_51766_MOESM1_ESM.pdf]

Supplementary material

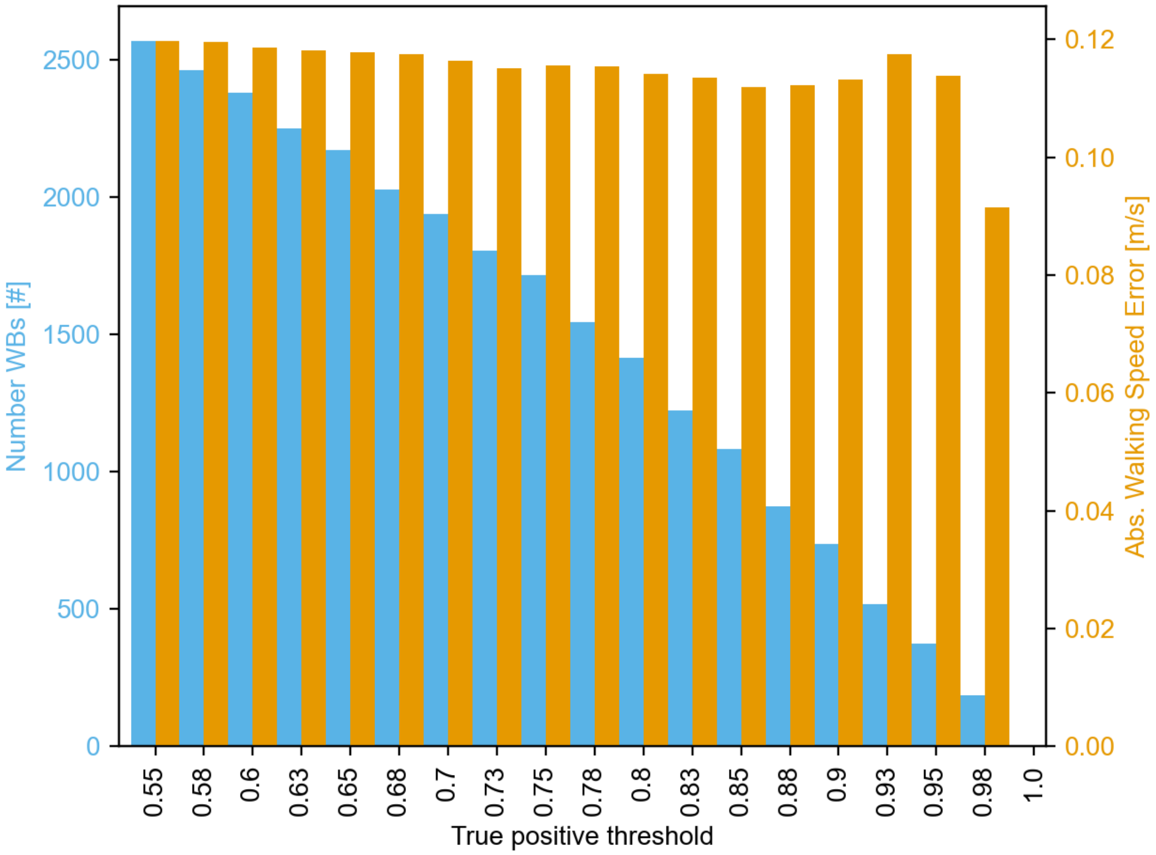

Supplementary Figure 1. Effect of selection of the threshold for identification of true-positive walking bouts (i.e. overlapping between walking bouts correctly identified by the wearable device and reference system) on the number of real-world walking bouts (WBs) detected as true-positive and included in analysis and the average absolute gait speed error across them.

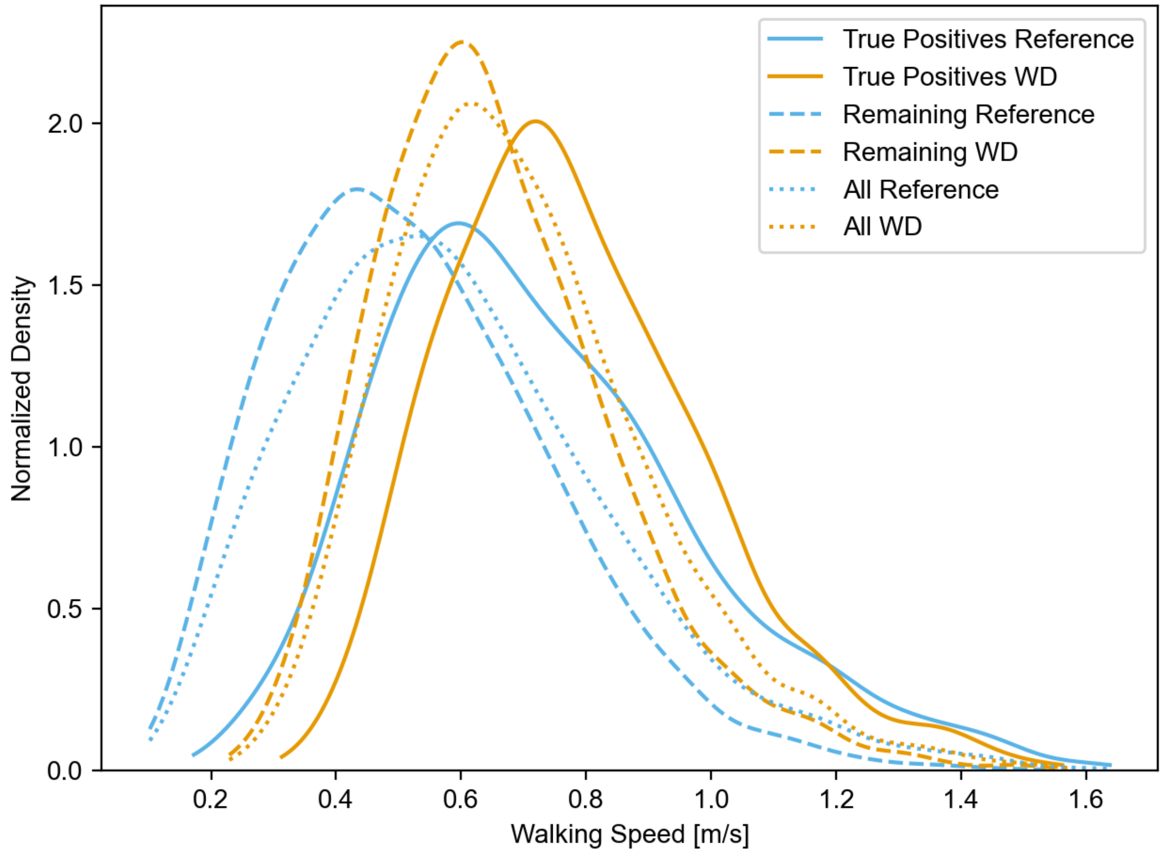

Supplementary Figure 2. Gait speed distributions overall considering all walking bouts (WBs) walking bouts, only true-positive walking bouts, and only false positive walking bouts. Comparing the reference system to the wearable device (WD). Each kernel density estimation is normalised to an area of 1 independently.

Supplementary Table 1. Results from the analysis only considering true positive walking bouts for cadence.

	Cohort	Reference [steps/min]	Wearable device [steps/min]	Error with LOA [steps/min]	Rel. Error with LOA [%]	Abs Error [steps/min]	Rel. Abs. Error [%]	ICC
Laboratory	CHF	99.13 [78.69 124.18]	95.78 [77.49 120.02]	3.35 [-7.69 14.38]	3.00 [-8.14 14.14]	4.33 [0.37 15.18]	4.27 [0.39 13.18]	0.90 [0.85, 0.94]
	COPD	97.88 [75.53 116.11]	94.94 [73.57 110.66]	2.95 [-3.57 9.46]	2.89 [-3.34 9.12]	3.38 [0.33 9.48]	3.38 [0.36 8.60]	0.93 [0.90, 0.95]
	HA	99.32 [74.96 117.23]	94.81 [74.62 110.52]	4.51 [-6.59 15.60]	4.28 [-6.08 14.64]	4.94 [0.33 14.36]	4.77 [0.41 14.43]	0.81 [0.73, 0.87]
	MS	94.64 [66.17 116.06]	93.86 [67.77 113.95]	0.78 [-15.69 17.25]	0.24 [-16.03 16.50]	4.04 [0.14 12.68]	4.27 [0.15 13.75]	0.83 [0.77, 0.87]
	PD	92.86 [68.62 115.40]	92.77 [71.24 109.90]	0.09 [-9.02 9.20]	-0.22 [-10.77 10.34]	3.38 [0.30 10.27]	3.76 [0.34 10.13]	0.93 [0.90, 0.95]
	PFF	96.64 [69.81 122.09]	95.73 [70.75 114.29]	0.92 [-14.68 16.51]	0.17 [-16.79 17.13]	3.97 [0.20 10.93]	4.08 [0.22 11.52]	0.85 [0.79, 0.89]
	All	96.23 [70.44 118.73]	94.45 [71.24 113.76]	1.78 [-11.20 14.76]	1.39 [-11.99 14.77]	3.96 [0.24 11.48]	4.08 [0.24 12.70]	0.87 [0.85, 0.89]
Real-world	CHF	98.76 [75.34 121.84]	98.04 [76.99 123.03]	0.73 [-13.99 15.44]	0.42 [-13.64 14.48]	4.22 [0.10 15.56]	4.18 [0.11 17.34]	0.87 [0.82, 0.90]
	COPD	87.45 [71.59 104.46]	85.53 [72.73 99.86]	1.92 [-10.32 14.17]	1.82 [-11.95 15.59]	4.32 [0.21 12.81]	4.91 [0.27 14.40]	0.77 [0.72, 0.81]
	HA	91.86 [71.46 114.15]	89.97 [72.21 111.61]	1.89 [-10.45 14.23]	1.59 [-11.76 14.95]	4.35 [0.22 14.39]	4.73 [0.23 16.73]	0.87 [0.84, 0.89]
	MS	88.29 [62.03 109.10]	89.66 [66.78 108.42]	-1.36 [-13.91 11.18]	-2.45 [-20.68 15.77]	4.23 [0.20 12.52]	5.38 [0.19 19.11]	0.88 [0.85, 0.91]
	PD	92.66 [67.85 111.56]	93.67 [76.86 109.11]	-1.01 [-13.70 11.67]	-1.77 [-17.73 14.19]	3.80 [0.11 16.28]	4.56 [0.11 22.21]	0.84 [0.80, 0.88]
	PFF	89.63 [64.31 113.62]	88.59 [65.79 110.87]	1.04 [-18.32 20.40]	0.37 [-17.93 18.67]	5.17 [0.14 16.74]	5.40 [0.16 19.14]	0.77 [0.70, 0.83]
	All	91.04 [69.61 113.92]	90.22 [71.27 111.23]	0.81 [-13.07 14.70]	0.35 [-15.21 15.90]	4.33 [0.14 14.93]	4.85 [0.16 17.18]	0.85 [0.83, 0.86]

Supplementary Table 2. Results from the analysis only considering true positive walking bouts for stride length.

	Cohort	Reference [m]	Wearable device [m]	Error with LOA [m]	Rel. Error with LOA [%]	Abs Error [m]	Rel. Abs. Error [%]	ICC
Laboratory	CHF	1.12 [0.68, 1.53]	1.08 [0.82, 1.43]	-0.03 [-0.36, 0.29]	-0.34 [-34.33, 33.64]	0.12 [0.01, 0.36]	11.96 [0.85, 34.36]	0.72 [0.57, 0.82]
	COPD	1.08 [0.82, 1.30]	1.12 [0.87, 1.39]	0.04 [-0.16, 0.24]	4.90 [-21.18, 30.98]	0.08 [0.01, 0.21]	8.50 [0.78, 22.49]	0.78 [0.69, 0.84]
	HA	1.04 [0.74, 1.28]	1.07 [0.76, 1.35]	0.03 [-0.23, 0.29]	4.17 [-26.87, 35.21]	0.10 [0.01, 0.25]	10.84 [0.77, 29.49]	0.72 [0.61, 0.80]
	MS	1.04 [0.70, 1.36]	1.08 [0.83, 1.39]	0.05 [-0.29, 0.38]	7.13 [-34.06, 48.31]	0.14 [0.01, 0.35]	15.11 [0.96, 46.39]	0.58 [0.47, 0.67]
	PD	1.02 [0.65, 1.35]	1.06 [0.81, 1.37]	0.04 [-0.30, 0.38]	7.71 [-47.94, 63.36]	0.13 [0.01, 0.32]	16.05 [1.24, 40.23]	0.65 [0.53, 0.73]
	PFF	0.86 [0.47, 1.38]	0.93 [0.70, 1.34]	0.06 [-0.24, 0.37]	14.70 [-48.91, 78.32]	0.13 [0.01, 0.33]	20.51 [0.90, 64.43]	0.76 [0.67, 0.82]
	All	1.02 [0.61, 1.37]	1.06 [0.76, 1.37]	0.04 [-0.27, 0.34]	7.13 [-38.87, 53.14]	0.12 [0.01, 0.33]	14.31 [0.86, 42.72]	0.72 [0.68, 0.75]
Real-world	CHF	1.14 [0.65, 1.67]	1.12 [0.80, 1.60]	-0.02 [-0.42, 0.37]	2.33 [-49.75, 54.42]	0.15 [0.02, 0.41]	15.91 [1.39, 71.58]	0.74 [0.67, 0.80]
	COPD	0.88 [0.55, 1.25]	1.05 [0.78, 1.37]	0.16 [-0.20, 0.53]	23.65 [-40.08, 87.38]	0.18 [0.01, 0.54]	25.37 [1.35, 91.30]	0.31 [0.21, 0.41]
	HA	0.92 [0.57, 1.34]	0.99 [0.68, 1.37]	0.08 [-0.22, 0.38]	11.70 [-37.30, 60.71]	0.12 [0.01, 0.38]	15.88 [1.05, 56.66]	0.74 [0.69, 0.78]
	MS	0.91 [0.58, 1.33]	1.02 [0.77, 1.37]	0.11 [-0.30, 0.51]	17.09 [-50.14, 84.33]	0.16 [0.01, 0.50]	21.59 [1.08, 79.37]	0.41 [0.28, 0.52]
	PD	0.97 [0.53, 1.42]	1.07 [0.70, 1.46]	0.09 [-0.33, 0.51]	15.57 [-53.96, 85.09]	0.16 [0.01, 0.51]	21.38 [0.76, 71.32]	0.53 [0.43, 0.63]
	PFF	0.88 [0.55, 1.24]	0.94 [0.72, 1.19]	0.05 [-0.33, 0.44]	11.61 [-49.74, 72.96]	0.15 [0.01, 0.41]	20.52 [1.38, 70.04]	0.42 [0.29, 0.54]
	All	0.94 [0.56, 1.38]	1.03 [0.73, 1.41]	0.09 [-0.30, 0.47]	14.60 [-46.65, 75.84]	0.15 [0.01, 0.48]	20.15 [1.08, 75.90]	0.59 [0.55, 0.62]
